# *Houttuynia cordata* Thunb. and its bioactive compound 2-undecanone significantly suppress benzo(a)pyrene-induced lung tumorigenesis by activating the Nrf2-HO-1/NQO-1 signaling pathway

**DOI:** 10.1186/s13046-019-1255-3

**Published:** 2019-06-07

**Authors:** Yanmei Lou, Zhenzhen Guo, Yuanfeng Zhu, Muyan Kong, Rongrong Zhang, Linlin Lu, Feichi Wu, Zhongqiu Liu, Jinjun Wu

**Affiliations:** 1 Joint Laboratory for Translational Cancer Research of Chinese Medicine of the Ministry of Education of the People’s Republic of China, International Institute for Translational Chinese Medicine, Guangzhou University of Chinese Medicine, Guangzhou, 510006 Guangdong China; 2 State Key Laboratory of Quality Research in Chinese Medicine, Macau University of Science and Technology, Macau, SAR China; 3 Hunan Zhengqing Pharmaceutical Group Limited, Huaihua, 418005 China

**Keywords:** *Houttuynia cordata* Thunb., 2-undecanone, benzo(a)pyrene, reactive oxygen species, DNA damage, inflammation, nuclear factor E2-related factor-2

## Abstract

**Background:**

Lung cancer remains the most common cause of cancer-related deaths, with a high incidence and mortality in both sexes worldwide. Chemoprevention has been the most effective strategy for lung cancer prevention. Thus, exploring novel and effective candidate agents with low toxicity for chemoprevention is essential and urgent. *Houttuynia cordata* Thunb. (Saururaceae) (*H. cordata*), which is a widely used herbal medicine and is also popularly consumed as a healthy vegetable, exhibits anti-inflammatory, antioxidant and antitumor activity. However, the chemopreventive effect of *H. cordata* against benzo(a)pyrene (B[a]P)-initiated lung tumorigenesis and the underlying mechanism remain unclear.

**Methods:**

A B[a]P-stimulated lung adenocarcinoma animal model in A/J mice *in vivo* and a normal lung cell model (BEAS.2B) *in vitro* were established to investigate the chemopreventive effects of *H. cordata* and its bioactive compound 2-undecanone against lung tumorigenesis and to clarify the underlying mechanisms.

**Results:**

*H. cordata* and 2-undecanone significantly suppressed B[a]P-induced lung tumorigenesis without causing obvious systemic toxicity in mice *in vivo*. Moreover, *H. cordata* and 2-undecanone effectively decreased B[a]P-induced intracellular reactive oxygen species (ROS) overproduction and further notably protected BEAS.2B cells from B[a]P-induced DNA damage and inflammation by significantly inhibiting phosphorylated H2A.X overexpression and interleukin-1β secretion. In addition, *H. cordata* and 2-undecanone markedly activated the Nrf2 pathway to induce the expression of the antioxidative enzymes heme oxygenase-1 (HO-1) and NAD(P)H: quinone oxidoreductase 1 (NQO-1). Nrf2 silencing by transfection with Nrf2 siRNA markedly decreased the expression of HO-1 and NQO-1 to diminish the reductions in B[a]P-induced ROS overproduction, DNA damage and inflammation mediated by *H. cordata* and 2-undecanone.

**Conclusions:**

*H. cordata* and 2-undecanone could effectively activate the Nrf2-HO-1/NQO-1 signaling pathway to counteract intracellular ROS generation, thereby attenuating DNA damage and inflammation induced by B[a]P stimulation and playing a role in the chemoprevention of B[a]P-induced lung tumorigenesis. These findings provide new insight into the pharmacological action of *H. cordata* and indicate that *H. cordata* is a novel candidate agent for the chemoprevention of lung cancer.

**Electronic supplementary material:**

The online version of this article (10.1186/s13046-019-1255-3) contains supplementary material, which is available to authorized users.

## Background

Worldwide, lung cancer remains the most frequently diagnosed cancer and the leading cause of cancer-related death, resulting in large social and economic burdens [1]. Many advanced treatments, including medical, surgical and radiotherapeutic interventions, have provided little effective improvement in the survival rates of patients diagnosed with primary lung malignancies [2]. The strong link between cigarette smoking and the development of lung cancer has been known for decades. The risk of lung cancer is 6 to 10 times higher in smokers than in nonsmokers [3], and almost 90% of patients diagnosed with lung cancer are cigarette smokers [4, 5]. Although it is widely recognized that smoking prevention and cessation are the best approaches to prevent lung cancer, tobacco-related lung carcinogenesis is still prevalent because of the difficulty in controlling smoking [2]. According to the WHO guidelines, chemoprevention has been the most effective strategy for lung cancer prevention, especially for smokers with existing pulmonary premalignancies [5, 6]. Therefore, it is essential and urgent to explore dietary factors that have the potential to prevent lung tumorigenesis.

Benzo(a)pyrene (B[a]P), which accounts for 22.5-69.8% of tobacco metabolites, can induce cell proliferation, inflammation, DNA alteration, and apoptosis, leading to lung cancer [7]. An evidenced-based study revealed that long-term exposure to B[a]P at a low dose could increase tumor incidence by up to 96.0% in animal models [8]. In part of its carcinogenic mechanism, B[a]P is metabolized into epoxide, which induces DNA adduct formation and causes mutations [9]. In addition, B[a]P-induced excessive production of reactive oxygen species (ROS), which results in oxidative stress, can lead to severe damage to DNA structure and is a prerequisite for B[a]P-associated tumorigenesis [10–12]. Additionally, oxidative stress-induced inflammation is one of the major contributors to lung cancer initiation and progression [13, 14]. ROS in the respiratory system can increase the levels of pulmonary inflammation mediators and then initiate or promote lung carcinogenesis [15]. More importantly, there is a close relationship between ROS-induced DNA damage and inflammation, which can together accelerate the process of lung tumorigenesis. On the one hand, DNA damage may lead to chronic inflammation, which has been associated with the development of lung cancer [16–18]. On the other hand, under inflammatory conditions, ROS are generated in inflammatory and epithelial cells and further cause oxidative and nitrative DNA damage [19, 20]. Therefore, the use of appropriate preventive pharmacological interventions that can effectively reduce B[a]P-induced DNA damage and inflammation has become an important strategy to prevent lung tumorigenesis.

Induction of enzymes that promote the detoxication of chemical carcinogens has been a broadly effective method for the chemoprevention of experimental carcinogenesis in rodent models [21]. The nuclear factor-erythroid 2-related factor 2 (Nrf2) signaling pathway has been targeted for the prevention of chemical carcinogenesis.Nrf2 has been identified as a key regulator of the inducible expression of antioxidative enzymes, anti-inflammatory proteins and conjugation/detoxification proteins, including NAD(P)H: quinone oxidoreductase 1 (NQO1) and heme oxygenase-1 (HO-1) [21]. Upon oxidative stress caused by B[a]P or pharmacologic induction, the Nrf2 pathway can be rapidly activated to counteract intracellular ROS generation, thereby attenuating DNA damage and inflammation promoted by B[a]P stimulation and contributing to reduced risk of mutation and subsequent lung tumorigenesis [21, 22]. Natural products are well known to exert their protective effects by removing free radicals, modulating the antioxidant defense system and performing carcinogen detoxification. To date, numerous compounds from natural plants have been found to activate Nrf2 signaling to effectively prevent B[a]P-induced lung tumorigenesis. These Nrf2 stimulators include sulforaphane, green tea phenols, curcumin, quercetin, catechin, and naringenin [23–28]. Unfortunately, most of the antioxidants have failed phase III clinical trials for chemoprevention because of their poor bioavailability and reproducibility and/or harmful outcomes [29]. Thus, exploring more novel and effective candidate agents with low toxicity for chemoprevention is essential and urgent.

*Houttuynia cordata* Thunb. (Saururaceae) (*H. cordata*) is a well-known traditional Chinese medicinal herb widely used in China and Japan. Growing evidence suggests that *H. cordata* performs a variety of pharmacological functions, including antiviral, antitumor, antileukemia, anti-inflammatory, antioxidant, and antimutagenic functions [30, 31]. Furthermore, *H. cordata* is also popularly consumed as a healthy vegetable in East Asia [32]. Chemically, *H. cordata* is composed of flavonoids, alkaloids, volatile oils, sterols, fatty acids, and polyphenolic acids [33, 34]. Among the components, 2-undecanone is a vital bioactive compound that exhibits various types of biological activity [35, 36] and is used as a standard marker for quality control of *H. cordata* in the Chinese Pharmacopoeia. According to modern pharmacological studies and clinical practices, *H. cordata* is especially suitable for the treatment of lung diseases. For example, during an outbreak of severe acute respiratory syndrome, *H. cordata* was proposed to be an effective alternative treatment [30]. *H. cordata* also exerts a protective effect against bleomycin-induced pulmonary fibrosis [30] and may alleviate lipopolysaccharide-induced lung inflammatory injury [37]. It has also been demonstrated that *H. cordata* exhibits anti–lung cancer activity [38]. However, the chemopreventive effect of *H. cordata* against B[a]P-induced lung carcinogenesis and the underlying mechanism have not yet been explored.

In the current study, a B[a]P-stimulated lung adenocarcinoma animal model in A/J mice *in vivo* and a normal lung cell (BEAS.2B) model *in vitro* were established to investigate the chemopreventive effects of *H. cordata and* 2-undecanone against B[a]P-induced lung tumorigenesis. We also clarified the underlying mechanisms by which *H. cordata* and 2-undecanone could prevent lung carcinogenesis by measuring molecular markers of oxidative DNA damage and inflammation mediated by the Nrf2-HO-1/NQO-1 signaling pathway. The results of this study will enhance understanding of the chemopreventive effects of *H. cordata* against B[a]P-stimulated lung tumorigenesis and the underlying mechanisms, thereby supporting the use of this novel candidate agent for the chemoprevention of lung cancer.

## Materials and methods

### Chemicals and reagents

A dried *Houttuyniae* Herba, the aerial part of *H. cordata*, was purchased from Hunan Zhengqing Pharmaceutical Co., Ltd. (Hunan, China), and was authenticated by Guangzhou University of Chinese Medicine as the aerial part of *Houttuynia cordata* Thunb. (Saururaceae). A voucher specimen was deposited in Guangzhou University of Chinese Medicine. 2-Undecanone (purity > 99%), benzo(a)pyrene (B[a]P, purity > 98%), tert-Butylhydroquinone (tBHQ), N-Acetyl-L-cysteine (NAC), and 3-(4, 5-dimethylthiazol-2-yl)-2, 5-diphenyltetrazolium bromide (MTT) were bought from Sigma-Aldrich (St. Louis, MO, USA). Primary antibodies NQO-1, HO-1 and Nrf2 were purchased from Abcam Inc. (Cambridge, MA, USA). Primary antibodies phosphorylated H2A.X (p-H2A.X) and Cyclin D1 were purchased from Cell Signaling Technology Inc. (Boston, USA). Primary antibodies interleukin-1β (IL-1β) and pro-IL-1β were purchased from R&D Systems Inc. (Minneapolis, Minnesota, USA). 5-Ethynyl-2’-deoxyuridine (EdU) kit, Nrf2-specific siRNA (siNrf2), control siRNA (siCtrl) and riboFECTTM CP Transfection kit were purchased from RiboBio Co., Ltd. (Guangzhou, China). Cellular ROS Detection Reagent (CM-H2DCFDA) was purchased from Invitrogen (Thermo Fisher Scientific Inc., Waltham, MA, US). Mouse IL-1β ELISA Kit was purchased from MultiSciences(Lianke)Biotech Co., Ltd. (Hangzhou, China). Anti-Rabbit HRP-DAB Cell & Tissue Staining Kit was purchased from R&D Systems Inc. (Minneapolis, Minnesota, USA). NE-PER nuclear and cytoplasmic extraction reagents were purchased from Pierce Biotechnology Inc. (Rockford, IL, USA). All other chemicals and solvents were of analytical grade or better and used as received.

### Preparation of *H.cordata* water extract and standardization

*H. cordata* was cut into pieces approximately 1 cm long. A total of 100 g of *H. cordata* was subjected to hydrodistillation for 4 h in a Clevenger apparatus following the procedure described in the Chinese Pharmacopoeia. The supernatant essential oils were isolated. The lower aqueous solution was filtered out with gauze. Next, the dregs were re-extracted under the same conditions. Afterward, the two successively obtained aqueous solutions were merged and condensed by a rotatory evaporator (EYELA, Rikakikai Co., Ltd., Tokyo, Japan) under reduced pressure. After cooling, the condensed filtrates and the two isolated supernatant essential oil samples were merged. The final concentration of the *H. cordata* water extract was 1 g/mL, and it was stored at -20 °C for later use.

Chemical profiling and standardization of *H.cordata* water extract using 2-undecanone was performed by using gas chromatography with flame ionization detection (GC–FID) with an external standard method (Additional materials and methods and Additional file 1: Figure S1). 2-Undecanone was measured in *H.cordata* water extract at mean levels of 0.099 ± 0.011 mg/g.

### Cell culture

BEAS-2B cells were obtained from ATCC (Manassas, Virginia, USA). The cells cultured in Bronchial Epithelial Cell Basal Medium (BEGM) medium supplemented with 0.4% (v/v) bovine pituitary extraction (BPE), 0.1% (v/v) hydrocortisone, 0.1% (v/v) human epidermal growth factor (hEGF), 0.1% (v/v) epinephrine, 0.1% (v/v) insulin, 0.1% (v/v) transferrin, 0.1% (v/v) triiodothyronine, 0.1% (v/v) retinoic acid, and 0.1% (v/v) gentamicin amphotericin-B (GA). The cells were grown at 37 °C in a humidified atmosphere with 5% CO_2_.

### Animals and treatments

Male A/J mice (4-6 weeks old, 18-22 g) were supplied by the Jackson Laboratory Animal Center (No.99612800000100) (Maine, USA). All mice were kept in the animal facility in a specific pathogen-free (SPF) animal laboratory (license number: SYXK (GZ) 2014-0144) at the International Institute for Translational Chinese Medicine, Guangzhou University of Chinese Medicine (Guangzhou, China). The mice were intraperitoneally injected with B[a]P (100 mg/kg). After two weeks, all mice were randomly divided into 6 groups (8 mice per group), as follows: a control group treated with B[a]P in a water vehicle, groups treated with 25 and 50 g/kg *H. cordata*, a control group treated with B[a]P in a sterilized corn oil vehicle, and groups treated with 100 and 200 mg/kg 2-undecanone. The mice were orally treated with the drugs five times a week for 38 weeks. The body weights were recorded every week. At the end of the treatment, the mice were sacrificed, and the organs were removed and weighed. The organ indexes (organ weight/body weight) were statistically analyzed. Mouse lung images were captured by using an M165C stereoscopic microscope (Leica Microsystems, Wetzlar, Germany). The lungs and plasma of the mice were collected for subsequent assays. Animal experiments were approved by the Guangzhou University of Chinese Medicine Animal Care and Use Committee (Guangzhou, China), and conducted in accordance with the ethical standards and national guidelines.

### MTT assay

BEAS-2B cells were seeded in 96-well plates for 24 h before the addition of drugs. The cells were exposed to vehicle (water), B[a]P (0-50 μM) alone, *H.cordata* (0-200 mg/mL) alone, B[a]p (5 μM) plus *H.cordata* (0-100 mg/mL), 2-undecanone (0-200 μM) alone, or B[a]p (5 μM) plus 2-undecanone (0-200 μM) for 48 h. At the end of the incubation, 100 μL of MTT solution (0.5 mg/mL in PBS) was added to each well, and the cells were incubated at 37 °C for an additional 4 h. Then, the medium was removed, the intracellular formazan was solubilized with 150 μL of DMSO, and the absorbance was read at 570 nm using a Victor X3 microplate reader (PerkinElmer, Waltham, MA, USA). The percentage of cell viability was calculated based on the measured absorbance relative to the absorbance of the control cells.

### 5-Ethynyl-2’-deoxyuridine (EdU) assay

EdU was used to detect cell proliferation ability. BEAS-2B cells were seeded into 96-well plates and exposed to vehicle (water), B[a]P (5 μM) alone, B[a]P (5 μM) plus *H.cordata* (12.5, 25, or 50 mg/mL), or B[a]P (5 μM) plus 2-undecanone (25, 50, or 100 μM) for 48 h. After incubation, the cells were incubated with EdU labeling medium (10 μM) for 24 h, fixed with 4% paraformaldehyde for 30 min, and incubated with glycine (2 mg/ml) for 5 min. Then, the cells were incubated with 1× Apollo 488 working solution for 30 min, washed with PBS, and stained with Hoechst 33342 dye for 30 min. Images were captured by using a Leica3000B fluorescence microscope (Leica, Germany). The percentages of EdU-positive cells were calculated from 6 random fields.

### Cell cycle assay

BEAS-2B cells were seeded in 6-well plates and exposed to the vehicle (water), B[a]P (5 μM) alone, B[a]P (5 μM) plus *H.cordata* (12.5, 25, 50 mg/mL), or B[a]P (5 μM) plus 2-undecanone (25, 50, 100 μM) for 48 h, respectively. After the incubation, the cells were harvested, fixed with 70% cold ethanol at 4 °C overnight, and stained with propidium iodide (5 μg/mL) for 30 min. Cell cycle was detected by a flow cytometry (BD Biosciences, San Jose, CA) and were analyzed by FlowJo 7.6 software.

### Protein preparation and Western blot analysis

At the end of treatment, the total proteins in the treated cells and the lung of mice were extracted using the RIPA buffer containing a protease inhibitor cocktail. The nuclear protein extracts of the cells were also prepared using NE-PER nuclear and cytoplasmic extraction reagents (Rockford, IL, USA) according to the manufacturer’s instructions. The proteins in the cell-free supernatants were prepared as previously published procedure [39]. Protein concentrations were determined with a BCA estimation kit according to the manufacturer’s instructions. Western blotting was performed as previously described [40].

### Comet assay

A comet assay was used to detect DNA strand breaks at the single-cell level. BEAS-2B cells were seeded in 6-well plates and exposed to vehicle (water), B[a]P (5 μM) alone, B[a]P (5 μM) plus *H.cordata* (12.5, 25, or 50 mg/mL), or B[a]P (5 μM) plus 2-undecanone (25, 50, or 100 μM) for 48 h. After incubation, the cells were harvested and suspended in low–melting point agarose. After the cells were spread on normal–melting point agarose–coated slides, the slides were covered with a coverslip and left at 4 °C. After removing the coverslips, the slides were then immersed in cell lysis solution for 60 minutes at 4 °C. Then, the slides were transferred to an electrophoretic box and left for 60 minutes at 4°C. Electrophoresis was performed at 4 °C for 25 minutes. The slides were then washed and immersed in ethanol for another 20 minutes. Finally, the slides were stained with EB solution (20 μg/mL) and covered with coverslips. Images were captured by using a Leica3000B fluorescence microscope (Leica, Germany).

### Immunofluorescence

BEAS-2B cells were seeded on confocal dishes and exposed to the vehicle (water), B[a]P (5 μM) alone, B[a]P (5 μM) plus *H.cordata* (12.5, 25, or 50 mg/mL), or B[a]P (5 μM) plus 2-undecanone (25, 50, or 100 μM) for 48 h, respectively. After the incubation, the cells were fixed in cold methanol, permeabilized with 0.1% TritonX-100 and blocked with 5% bovine serum albumin. Then, the cells were incubated with a p-H2A.X (1:100) or a Nrf2 (1:100) antibody for 1 h and then stained with a secondary fluorescent antibody (1:200; Alexa Fluor 568, Abcam Inc. Cambridge, MA, USA). Finally, the cells were incubated with DAPI for another 20 min. Fluorescence signals were detected using a Leica TCS SP8 confocal fluorescence microscope (Leica, Germany). The relative fluorescence of p-H2A.X in the cells was analysed by ImageJ software.

### Intracellular ROS measurement

BEAS-2B cells were seeded in six-well plates and exposed to vehicle (water), B[a]P (5 μM) alone, B[a]P (5 μM) plus *H.cordata* (12.5, 25, or 50 mg/mL), or B[a]P (5 μM) plus 2-undecanone (25, 50, or 100 μM) for 48 h. N-Acetyl-L-cysteine (NAC, 2 mM), a well-known ROS inhibitor, was used as a positive control. After incubation, the cells were washed twice with PBS and incubated with a ROS detection reagent (CM-H2DCFDA, 5 μM) for 30 min at 37 °C. The fluorescence signals were detected by using flow cytometry (BD Biosciences, San Diego, CA, USA) or a Leica3000B fluorescence microscope (Leica, Germany). The results were analyzed with FlowJo 7.6 software.

### siRNA interference

siRNA interference was performed using a riboFECT^TM^ CP Transfection Kit (RiboBio Co., Ltd., Guangzhou, China) according to the manufacturer's instructions. Briefly, siRNA was diluted in transfection buffer and then mixed with riboFECT^TM^ CP. The mixture was incubated at room temperature for 0-10 minutes to form a transfection complex. The complex was added to a suitable amount of cell culture medium for transfection. Finally, the cells were transfected with 50 nM Nrf2-targeting siRNA (siNrf2) or control siRNA (siCon). After a 24 h transfection, the cells were exposed to the same treatment described above. At the end of treatment, the cells were collected for Western blot analysis, ROS content measurement or MTT assay under the same conditions described above.

### Immunohistochemistry

At the end of treatment, the lung tissues of the mice were removed, fixed in paraformaldehyde, embedded in paraffin, and sliced into 4 μm sections. Then, the slices were deparaffinized, rehydrated and incubated with sodium citrate for antigen retrieval. Next, the slides were rinsed in PBS and incubated with antibodies against p-H2A.X, NQO-1, HO-1 or Nrf2 at 4 °C overnight. The following steps were performed using an immunostaining kit (Boster Biological Technology Co., Ltd.) according to the manufacturer’s instructions. All sections were imaged under a microscope (Leica, Germany).

### Enzyme-linked immunosorbent assay (ELISA)

At the end of treatment, the plasma of the mice was collected. The levels of IL-1β were detected by a mouse IL-1β ELISA kit according to the manufacturer's protocol. The optical density was measured at 450 nm by using a microplate reader Victor X3 (PerkinElmer, Waltham, MA, USA).

### Hematoxylin and Eosin (H&E) Staining

At the end of treatment, lung tissues of the mice were removed and fixed in 4% paraformaldehyde, then embedded in paraffin and sliced into 4 μm. Following, The slices were dewaxed and rehydrated, then stained by hematoxylin for 2 min and 1 % ethyl hydrochloride for 5 s. Finally, the cytoplasm was stained by eosin for 2 min. The images were acquired by using a fluorescence inversion microscope (Leica, Germany).

### Data analysis

The data are expressed as the mean ± standard deviation (SD). The statistical significance of data was analyzed using Student’s *t*-test or one-way analysis of variance (ANOVA) by SPSS 19.0. Values of *p* < 0.05 was considered to be statistically significant.

## Results

### *H. cordata* and 2-undecanone prevent lung tumorigenesis in B[a]P-induced lung cancer mouse model

**Fig. 1:**
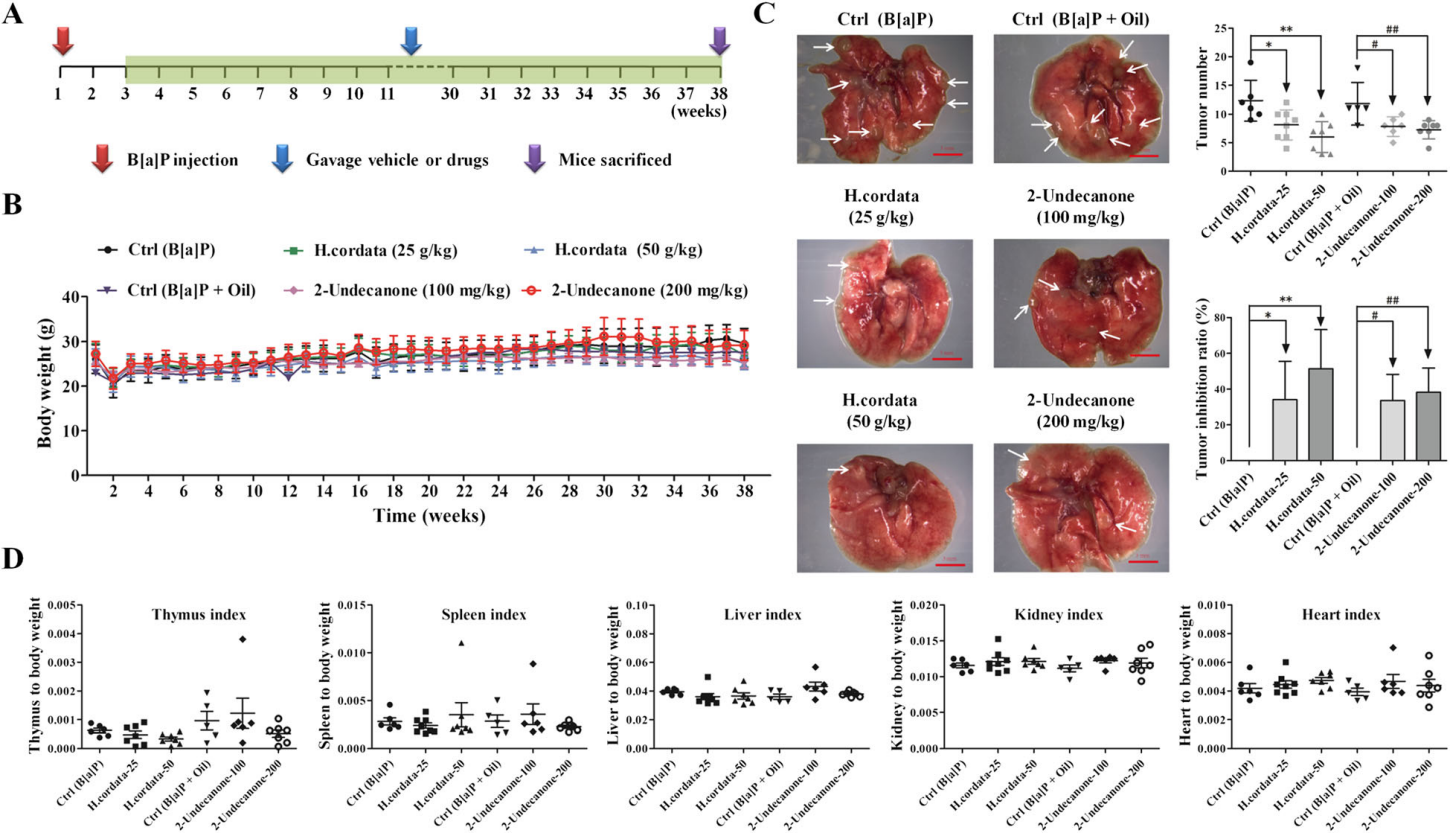
*H. cordata* and 2-undecanone prevent tumorigenesis in a B[a]P-induced lung cancer mouse model. **a** A/J mice were intraperitoneally injected with B[a]P (100 mg/kg) in the 1st week, orally treated with *H.cordata* (25 or 50 g/kg) or 2-undecanone (100 or 200 mg/kg) five times a week from the 3rd to the 38th week, and finally sacrificed after treatment. **b** Average body weight curves for the A/J mice (*n* = 8). **c** Photos of pulmonary nodules dissected from the A/J mice (scale bar: 3 mm). All photos were obtained using a stereoscopic microscope. Tumor numbers and tumor inhibition rates were analyzed. The data represent the mean ± SD (*n* = 8). **d** Organ indexes for A/J mouse hearts, livers, spleens, kidneys and thymuses. ^*^p < 0.05 and ^**^p < 0.01 compared with the control group (given B[a]P); ^#^*p* < 0.05 and ^##^*p* < 0.01 compared with the control group (given B[a]P + oil)

A B[a]P-induced lung cancer mouse model was established to evaluate the chemopreventive effects of *H. cordata* and 2-undecanone against lung tumorigenesis. As shown in Fig. 1a, A/J mice were intraperitoneally injected with B[a]P (100 mg/kg) at the 1st week. From the 3rd to 38th weeks, *H.cordata* (25 or 50 g/kg) or 2-undecanone (100 or 200 mg/kg) was administered by gavage five times a week. There were no significant body weight differences between the control group A/J mice (given only B[a]P) and the treatment group A/J mice during the experiment (Fig. 1b). Stereological observations and H&E staining of the pathological sections showed that the lung tissue was cancerous after the model was established, but the symptoms were relieved after treatment with *H. cordata* and 2-undecanone (Additional file 1: Figure S2). The results regarding visible tumors on the lung surface among the groups are shown in Fig. 1c. In contrast to the control mice (given B[a]P), which exhibited abundant tumor loci, the mice receiving B[a]P and *H. cordata* at 25 and 50 g/kg exhibited significantly (*p* < 0.05 and *p* < 0.01, respectively) decreased mean tumor numbers, and the corresponding tumor inhibition rates were 34.12 ± 21.42% (*p* < 0.05) and 51.35 ± 21.96% (*p* < 0.01), respectively (Fig. 1c). In contrast to the control mice (given B[a]P and oil) showing abundant tumor loci, the mice receiving B[a]P with 2-undecanone at 100 and 200 mg/kg also exhibited significantly (*p* < 0.05 and *p* < 0.01, respectively) decreased mean tumor numbers, and the corresponding tumor inhibition rates were 33.62 ± 14.60% (*p* < 0.05) and 38.26 ± 13.59% (*p* < 0.01), respectively (Fig. 1c). The organ indexes are displayed in Fig. 1d; no considerable differences were observed in the indexes for the heart, kidney, thymus, liver and spleen between the model group and the treatment groups.

### *H. cordata* and 2-undecanone protect BEAS-2B cells from B[a]P-induced cytotoxicity, proliferation inhibition and cell cycle arrest

**Fig. 2:**
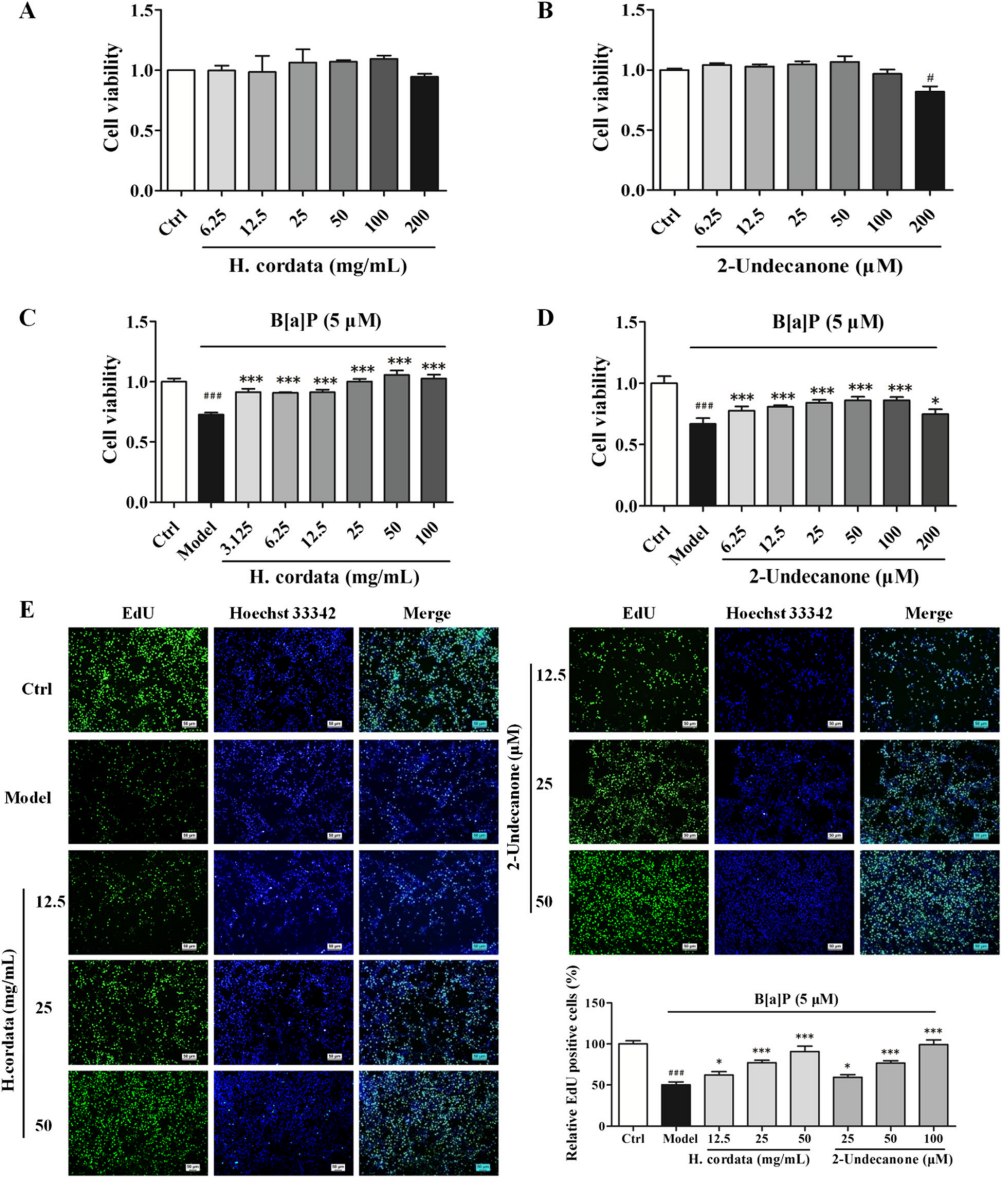
*H. cordata* and 2-undecanone protect BEAS-2B cells from B[a]P-induced cytotoxicity and proliferation inhibition. The cytotoxicity of *H. cordata* (6.25 to 200 mg/L, 48 h) (**a**) and 2-undecanone (6.25 to 200 μM, 48 h) (**b**) toward BEAS-2B cells was evaluated by using an MTT assay. The viability of BEAS.2B cells was also measured by MTT assay after treatment with *H. cordata* (3.125-100 mg/mL) (**c**) or 2-undecanone (6.25-200 μM) (**d**) for 48 h in the presence or absence of B[a]P (5 μM). **e** EdU incorporation was evaluated after treatment of cells with vehicle, B[a]P (5 μM) alone, B[a]P (5 μM) plus *H.cordata* (12.5, 25, or 50 mg/mL), or B[a]P (5 μM) plus 2-undecanone (25, 50, or 100 μM) for 48 h. The EdU-positive cells in each group were quantified as the percentage of those in the control group. The data represent the mean ± SD (n = 3). ^#^*p* < 0.05 and ^###^*p* < 0.001 compared with the control cells (given water); ^*^p < 0.05 and ^***^p < 0.001 compared with the model cells (given B[a]P)

The cytotoxicity of *H. cordata* and 2-undecanone toward BEAS-2B cells was evaluated by using an MTT assay. Compared with vehicle (water) treatment, treatment with B[a]P resulted in a significant decrease in the viability of BEAS-2B cells in a dose-dependent manner (Additional file 1: Figure S3, *p* < 0.05 or *p* < 0.001). Incubation of cells with *H. cordata* at concentrations from 6.25 to 200 mg/L or with 2-undecanone at concentrations from 6.25 to 200 μM for 48 h did not change or weakly decreased cell viability (Fig. 2a and b). However, in contrast to B[a]P (5 μM) treatment alone (in the model group), cotreatment with B[a]P and *H.cordata* (3.125-100 mg/mL) or 2-undecanone (6.25-200 μM) effectively increased cell viability in a dose-dependent manner (Fig. 2c and d, *p* < 0.05 or *p* < 0.001).

**Fig. 3:**
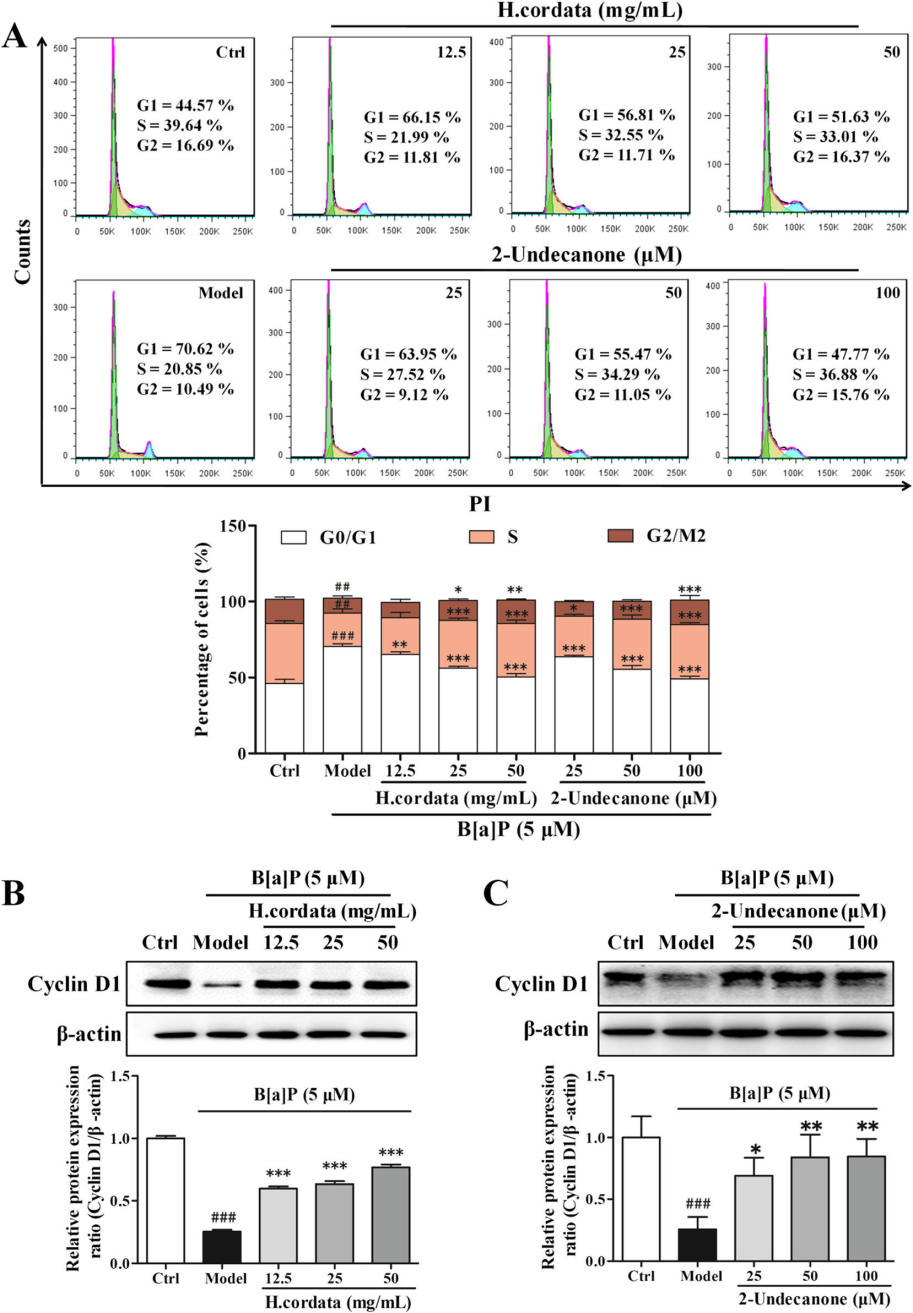
*H. cordata* and 2-undecanone protect BEAS-2B cells from B[a]P-induced cell cycle arrest. The cell cycle was examined by PI staining. The percentages of cell cycle arrest were analyzed by flow cytometry (**a**) and by analysis of the expression of cyclin D1 in cells after treatment with *H. cordata* (12.5, 25, or 50 mg/mL) (**b**) or 2-undecanone (25, 50, or 100 μM) (**c**) for 48 h in the presence or absence of B[a]P (5 μM). The data represent the mean ± SD (*n* = 3). ^##^*p* < 0.01 and ^###^*p* < 0.001 compared with the control cells (given water); ^*^p < 0.05, ^**^p < 0.01, and ^***^p < 0.001 compared with the model cells (given B[a]P)

Furthermore, EdU assays and cell cycle assays were performed to analyze the protective effects of *H. cordata* and 2-undecanone against B[a]P-induced cell proliferation inhibition. In contrast to the control cells (given water), which showed a relative abundance of EdU-positive cells, B[a]P (5 μM)-treated cells showed significantly suppressed cell proliferation rates (Fig. 2e, *p* < 0.001). However, compared with incubation with B[a]P alone (in the model group), incubation of cells with B[a]P plus *H. cordata* (12.5, 25 or 50 g/kg) or 2-undecanone (25, 50 or 100 μM) for 48 h markedly increased the proliferation rate in a dose-dependent manner (Fig. 2e, *p* < 0.05 or *p* < 0.001). In addition, B[a]P (5 μM) notably arrested the cell cycle at the G0/G1 phase by increasing the percentage of cells in this phase from 46.05 ± 2.75% (the control level) to 70.21 ± 1.65% (Fig. 3a, *p* < 0.01). However, cotreatment with B[a]P and *H. cordata* or 2-undecanone significantly decreased the percentage of cells arrested in G0/G1 phase in a dose-dependent manner compared with B[a]P treatment alone (in model cells) (Fig. 3a, *p* < 0.01 or *p* < 0.001). The levels of cyclin D1, a vital regulator required for G1 phase progression, were further determined by using Western blot analysis. It was observed that the suppression of cyclin D1 protein levels by B[a]P could be significantly reversed by *H. cordata* (Fig. 3b, *p* < 0.001) or 2-undecanone (Fig. 3c, *p* < 0.05 or *p* < 0.01) treatment.

### *H. cordata* and 2-undecanone protect BEAS-2B cells and A/J mice from B[a]P-induced DNA damage

**Fig. 4:**
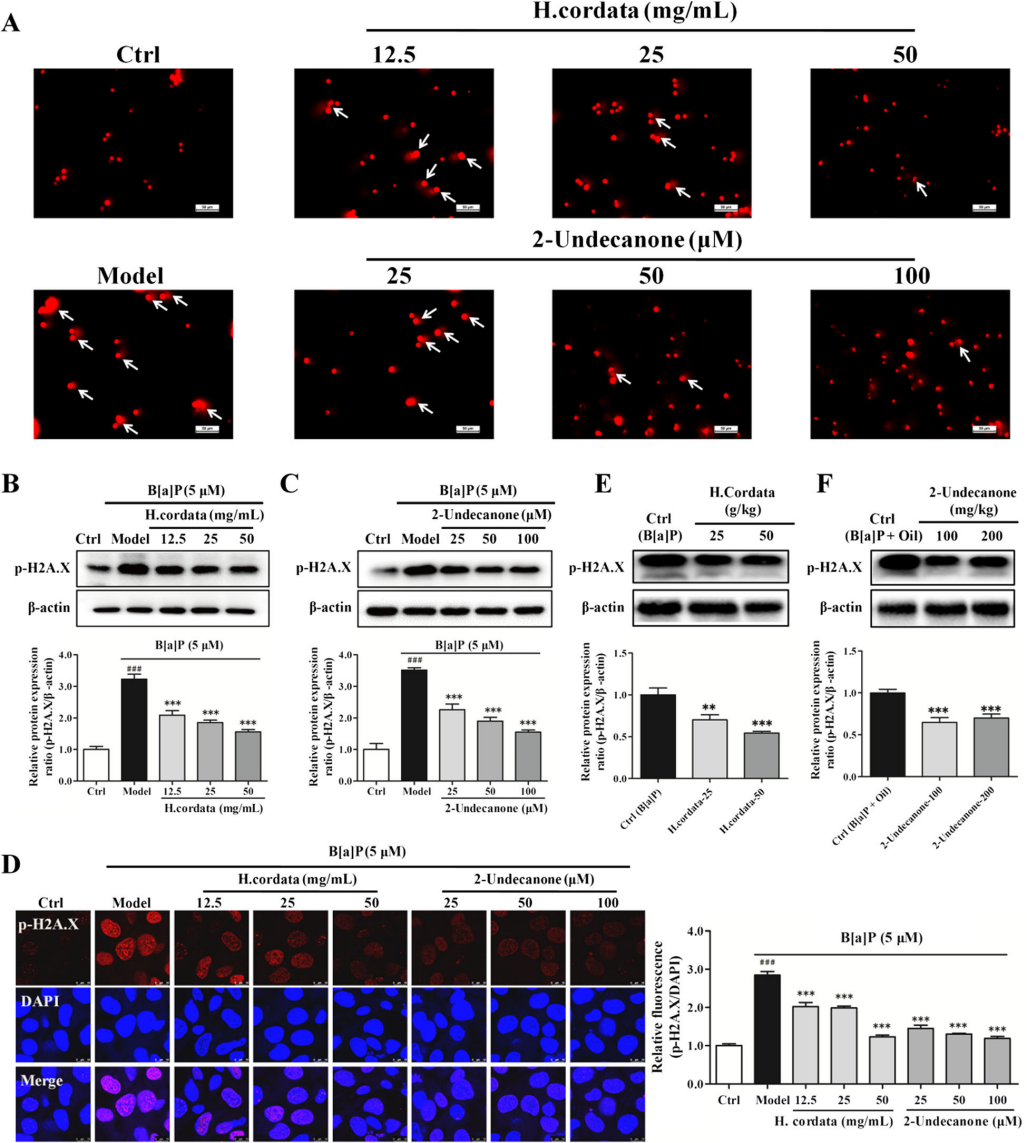
*H. cordata* and 2-undecanone protect BEAS-2B cells and A/J mice from B[a]P-induced DNA damage. **a** Representative comet tail images (scale bar: 50 μm) showing the DNA damage response after treatment with vehicle, B[a]P (5 μM) alone, B[a]P (5 μM) plus *H.cordata* (12.5, 25, or 50 mg/mL), or B[a]P (5 μM) plus 2-undecanone (25, 50, or 100 μM) for 48 h. The protein levels of the DNA damage marker p-H2A.X were detected in the cells after the same treatment regimens with *H. cordata* (**b**) or 2-undecanone (**c**) by Western blot analysis. **d** Representative confocal images (scale bar: 50 μm) of double-stained cells subjected to the described treatments and stained for p-H2A.X (red) and with DAPI (blue). **e** The protein levels of p-H2A.X were detected in mice after treatment with *H.cordata* (25 or 50 g/kg) (**e**) or 2-undecanone (100 or 200 mg/kg) (F) by Western blot analysis. The data represent the mean ± SD (*n* = 3). ^###^*p* < 0.001 compared with the control cells (given water); ^*^p < 0.05, ^**^p < 0.01, and ^***^p < 0.001 compared with the model cells (given B[a]P)

A comet assay was performed to detect the DNA damage rates in treated BEAS-2B cells. As shown in Fig. 4a, in contrast to the control cells (given water), cells treated with B[a]P (5 μM) for 48 h exhibited notably enhanced fluorescence in migrated DNA and extended tails of disrupted DNA fragments, suggesting that B[a]P could cause DNA damage in BEAS-2B cells. In contrast, compared with the B[a]P model group, the groups incubated with B[a]P plus*H. cordata* (12.5, 25 or 50 g/kg) or 2-undecanone (25, 50 or100 μM) for 48 h exhibited attenuated B[a]P-induced DNA damage, as demonstrated by decreased fluorescence and reduced migrated tails of damaged DNA.

DNA double-strand breaks induce histone H2A.X phosphorylation, which is associated with the recruitment of repair factors to damaged DNA. Thus, p-H2A.X has been used as a marker for DNA damage [41–43]. Hence, the protein levels of p-H2A.X in BEAS-2B cells and A/J mouse lung tissue were further determined by Western blot analysis. As shown in Fig. 4b, compared with control cells (given water), cells treated with B[a]P showed markedly increased p-H2A.X protein levels (*p* < 0.001). In contrast to B[a]P exposure alone (in the model group), exposure to B[a]P plus 12.5, 25 or 50 g/kg *H. cordata* significantly decreased the p-H2A.X protein levels in a dose-dependent manner (*p* < 0.001). Similar results were also observed after incubation with B[a]P plus 25, 50 or 100 μM 2-undecanone, which significantly decreased the B[a]P-induced high expression of p-H2A.X in a dose-dependent manner (Fig. 4c, *p* < 0.001). An immunofluorescence assay was further performed to confirm the protective effects of *H. cordata* and 2-undecanone against B[a]P-induced DNA damage. As shown in Fig. 4d, the immunofluorescence of p-H2A.X in the *H. cordata* and 2-undecanone treatment groups was strikingly lower than that in the B[a]P model group (*p* < 0.001), which was consistent with the p-H2A.X protein levels obtained from the Western blot analysis (Fig. 4b and c). The p-H2A.X protein levels in the lung tissues of mice were also determined. Both *H. cordata* and 2-undecanone treatment significantly downregulated p-H2A.X expression compared with B[a]P alone (Fig. 4e and f, *p* < 0.01 or *p* < 0.001).

### *H. cordata* and 2-undecanone protect BEAS-2B cells and A/J mice from B[a]P-induced inflammation

**Fig. 5:**
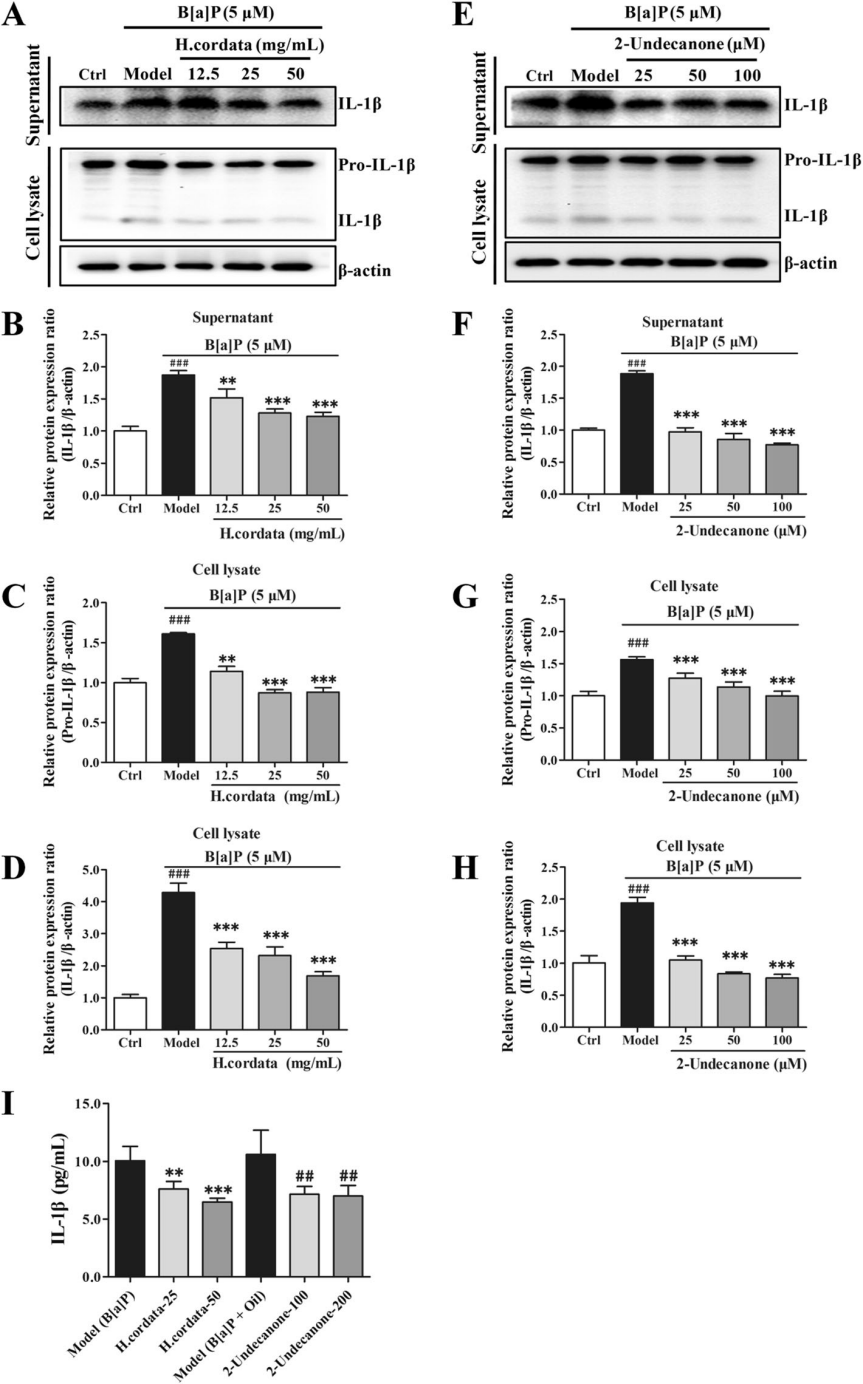
*H. cordata* and 2-undecanone protect BEAS-2B cells and A/J mice from B[a]P-induced inflammation. The protein levels were measured by using Western blot analysis. **a** and **b** The protein levels of IL-1β in the culture supernatants were detected after treatment with vehicle, B[a]P (5 μM) alone, or B[a]P (5 μM) plus *H.cordata* (12.5, 25, or 50 mg/mL) for 24 h. The protein levels of pro-IL-1β (**a** and **c**) and IL-1β (**a** and **d**) in the cell lysates were detected after the same *H.cordata* treatments. (**e** and **f**) The protein levels of IL-1β in the culture supernatants were detected after treatment with vehicle, B[a]P (5 μM) alone, or B[a]P (5 μM) plus 2-undecanone (25, 50, or 100 μM) for 24 h. The protein levels of pro-IL-1β (**e** and **g**) and IL-1β (E and H) in the cell lysates were detected after the same 2-undecanone treatments. **i** The IL-1β levels in the plasma of mice were detected with an ELISA kit. The data represent the mean ± SD (*n* = 3). ^##^*p* < 0.01 and ^###^*p* < 0.001 compared with the control cells (given water) or control mice (given B[a]P + oil); ^**^p < 0.01 and ^***^p < 0.001 compared with the model cells (given B[a]P) or control mice (given B[a]P)

The protein levels of related inflammatory markers in BEAS-2B cells were determined via Western blot analysis. The protein levels of IL-1β (Fig. 5a and b) in the culture supernatants and the protein levels of pro-IL-1β (Fig. 5a and c) and IL-1β (Fig. 5a and d) in the cell lysates of B[a]P-treated cells were markedly higher than those in the supernatants and lysates of control cells (given water), respectively (*p* < 0.001). In contrast to B[a]P exposure alone (in the model group), exposure to B[a]P plus 12.5, 25 or 50 g/kg *H. cordata* for 24 h significantly decreased the IL-1β protein levels in the culture supernatants (Fig. 5a and b, *p* < 0.01 or *p* < 0.001), and also significantly reduced the pro-IL-1β (Fig. 5a and c) and IL-1β (Fig. 5a and d) protein levels in the cell lysates in a dose-dependent manner (*p* < 0.01 or *p* < 0.001). Similar results were also observed for 2-undecanone, as incubation with B[a]P plus 25, 50 or 100 μM 2-undecanone for 24 h significantly attenuated the B[a]P-induced increases in IL-1β protein levels in the culture supernatants (Fig. 5e and f, *p* < 0.001), and also notably diminished the B[a]P-induced increases in pro-IL-1β (Fig. 5e and g) and IL-1β (Fig. 5e and h) protein levels in the cell lysates in a dose-dependent manner (*p* < 0.001). The IL-1β levels in the plasma of the mice were detected by ELISA. Both *H. cordata* and 2-undecanone treatment along with B[a]P significantly downregulated IL-1β levels compared with the control treatment (given B[a]P or B[a]P + oil) (Fig. 5i, *p* < 0.01 or *p* < 0.001).

### *H. cordata* and 2-undecanone decrease the B[a]P-induced over production of intracellular ROS

**Fig. 6:**
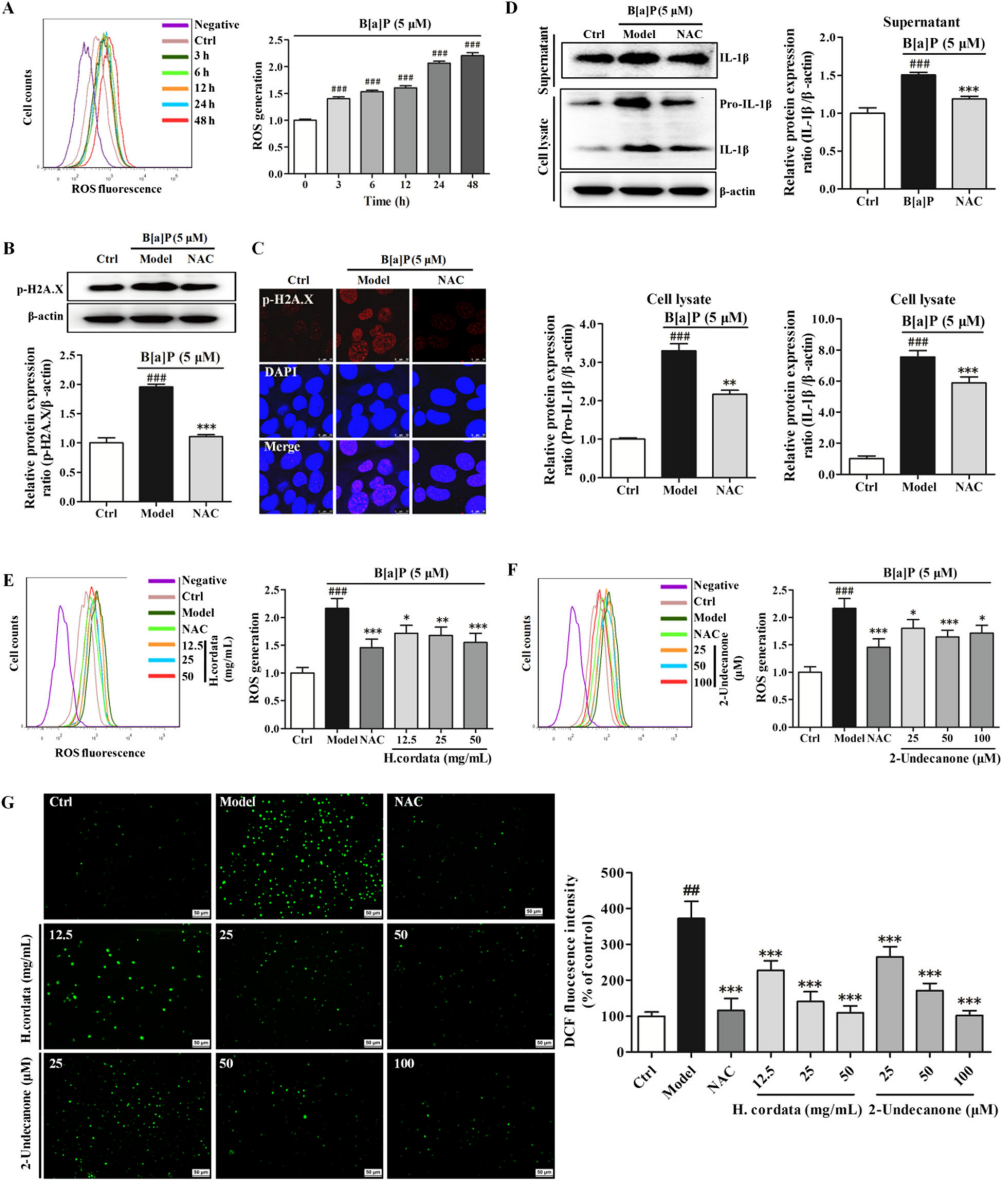
*H. cordata* and 2-undecanone decrease the overproduction of intracellular ROS induced by B[a]P. **a** Intracellular ROS levels were detected by flow cytometry after exposure to B[a]P (5 μM) for 0-48 h. **b** and **c** p-H2A.X expression in cells after treatment with vehicle, B[a]P (5 μM) alone, or B[a]P (5 μM) plus NAC (2 mM) for 48 h was detected by Western blot analysis and immunofluorescence analysis. **d** The protein levels of IL-1β in the culture supernatants and of pro-IL-1β and IL-1β in the cell lysates after the same treatments were detected by Western blot analysis. **e** and **f** Intracellular ROS levels were detected by flow cytometry after treatment with vehicle, B[a]P (5 μM) alone, B[a]P (5 μM) plus *H.cordata* (12.5, 25, or 50 mg/mL) or B[a]P (5 μM) plus 2-undecanone (25, 50, or 100 μM) for 48 h. **g** Intracellular ROS generation was detected by fluorescence microscopy (scale bar: 50 μm) after treatment with vehicle, B[a]P (5 μM) alone, B[a]P (5 μM) plus *H.cordata* (12.5, 25, or 50 mg/mL) or B[a]P (5 μM) plus 2-undecanone (25, 50, or 100 μM) for 48 h. The data represent the mean ± SD (*n* = 3). ^###^*p* < 0.001 compared with the control cells (given water); ^*^p < 0.05, ^**^p < 0.01, and ^***^p < 0.001 compared with the model cells (given B[a]P).

As shown in Fig. 6a, incubation of cells with B[a]P at 5 μM significantly increased intracellular ROS production in a time-dependent manner (*p* < 0.001). The results of Western blot analysis and immunofluorescence assays confirmed that incubation with B[a]P could significantly increase p-H2A.X expression (Fig. 6b and c, *p* < 0.001), while exposure to NAC (2 mM, 48h), a well-known ROS inhibitor, markedly reversed the B[a]P-induced p-H2A.X overexpression (Fig. 6b and c, *p* < 0.001). Exposure to NAC along with B[a]P also significantly attenuated the B[a]P-induced increases in pro-IL-1β and IL-1β protein levels in both the cell lysates and cell culture supernatants (Fig. 6d, *p* < 0.01 or *p* < 0.001). Thus, the results indicated that the B[a]P-induced overproduction of intracellular ROS could induce DNA damage and inflammation. The results from flow cytometry showed that exposure to *H. cordata* at 12.5, 25 or 50 g/kg for 48 h significantly decreased the B[a]P-mediated intracellular ROS overproduction in a dose-dependent manner (Fig. 6e, *p* < 0.05, *p* < 0.01 or *p* < 0.001) in the treated cells compared with the B[a]P model cells. Treatment with 2-undecanone at 25, 50 or 100 μM for 48 h also significantly reduced the intracellular ROS overproduction induced by B[a]P (Fig. 6f, *p* < 0.05 or *p* < 0.001). Intracellular ROS generation was also detected by fluorescence microscopy. Similar results were observed; B[a]P treatment increased intracellular ROS production compared with the control (water) treatment. However, incubation of B[a]P-treated cells with *H. cordata* or 2-undecanone notably diminished B[a]P-induced ROS overproduction (Fig. 6g). The correlation between ROS levels and DNA damage or inflammation was analyzed by using Pearson analysis. The results showed that the decrease in ROS levels was closely and positively related to the reductions in DNA damage (Additional file 1: Figure S4, *p* < 0.05) and inflammation (Additional file 1: Figure S5, *p* < 0.05) mediated by *H. cordata* and 2-undecanone.

### *H. cordata* and 2-undecanone activate the Nrf2 signaling pathway

**Fig. 7:**
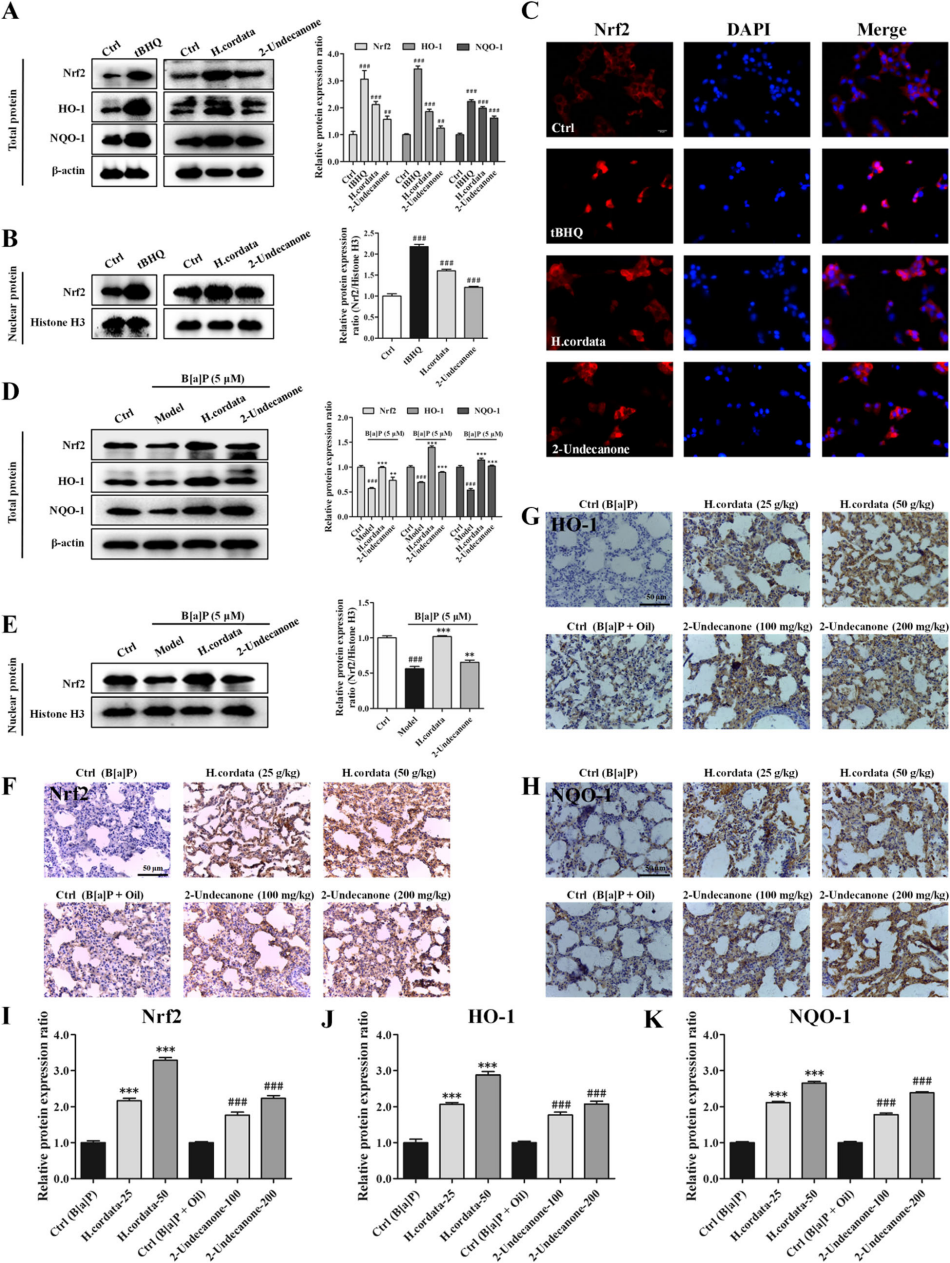
*H. cordata* and 2-undecanone activate the Nrf2 signaling pathway. The protein levels were measured by using Western blot analysis. Tertiary butylhydroquinone (tBHQ), a well-known Nrf2 activator, was used as a positive control. **a** The total protein levels of Nrf2, HO-1 and NQO-1 were measured in cells exposed to vehicle, tBHQ (10 μM), *H. cordata* (25 mg/mL) or 2-undecanone (50 μM). **b** The nuclear Nrf2 protein levels were measured in cells subjected to the same treatments. **c** Representative confocal images (scale bar: 50 μm) of double-stained cells subjected to the described treatments and stained for Nrf2 (red) and with DAPI (blue). **d** The total protein levels of Nrf2, HO-1 and NQO-1 were measured in cells exposed to vehicle, B[a]P (5 μM) alone, B[a]P (5 μM) plus *H.cordata* (25 mg/mL) or B[a]P (5 μM) plus 2-undecanone (50 μM). (E) The nuclear Nrf2 protein levels were measured in cells subjected to the same treatments. (F-K) The expression of Nrf2, HO-1 and NQO-1 in the lung tissues of mice after treatment was detected by immunohistochemistry. The data represent the mean ± SD (*n* = 3). ^**^p < 0.01 and ^***^p < 0.001 compared with the model cells (given B[a]P) or control mice (given B[a]P); ^###^*p* < 0.001 compared with the control cells (given water) or control mice (given B[a]P + oil)

The protein levels of Nrf2, HO-1 and NQO-1 in BEAS-2B cells and A/J mice were determined via Western blot analysis and immunohistochemistry, respectively. As shown in Fig. 7a, compared with those of control cells (given water), whole-cell lysates of cells incubated with *H. cordata* (25 g/kg) or 2-undecanone (50 μM) exhibited significantly increased protein levels of Nrf2, HO-1 and NQO-1 (*p* < 0.01 or *p* < 0.001). The same *H. cordata* or 2-undecanone treatment also resulted in significant increases in nuclear Nrf2 protein levels (Fig. 7b, *p* < 0.001). tBHQ, a known inducer of Nrf2, was used as a positive control and markedly upregulated the total and nuclear Nrf2 protein levels (Fig. 7a and b, *p* < 0.001). The immunofluorescence results also showed that both *H. cordata* and 2-undecanone notably promoted the translocation of Nrf2 from the cytoplasm to the nucleus (Fig. 7c). Furthermore, treatment with B[a]P at 5 μM significantly decreased the protein levels of Nrf2, HO-1 and NQO-1 in whole-cell lysates (Fig. 7d, *p* < 0.001) and decreased nuclear Nrf2 protein levels (Fig. 7e, *p* < 0.001). In contrast, treatment with *H. cordata* or 2-undecanone markedly attenuated the downregulation of Nrf2, HO-1 and NQO-1 expression induced by B[a]P (Fig. 7d and e, *p* < 0.01 or *p* < 0.001). Furthermore, immunohistochemistry was performed to detect the expression of Nrf2, HO-1 and NQO-1 in the lung tissues of mice. Compared with the control treatment (given B[a]P or B[a]P + oil), both *H. cordata* and 2-undecanone treatment strikingly enhanced the expression of Nrf2, HO-1 and NQO-1 in a dose-dependent manner (Fig. 7f-k, *p* < 0.001).

### Nrf2-HO-1/NQO-1 signaling pathway mediates the protective effects of *H. cordata* and 2-undecanone against B[a]P-induced DNA damage, inflammation and cytotoxicity

**Fig. 8:**
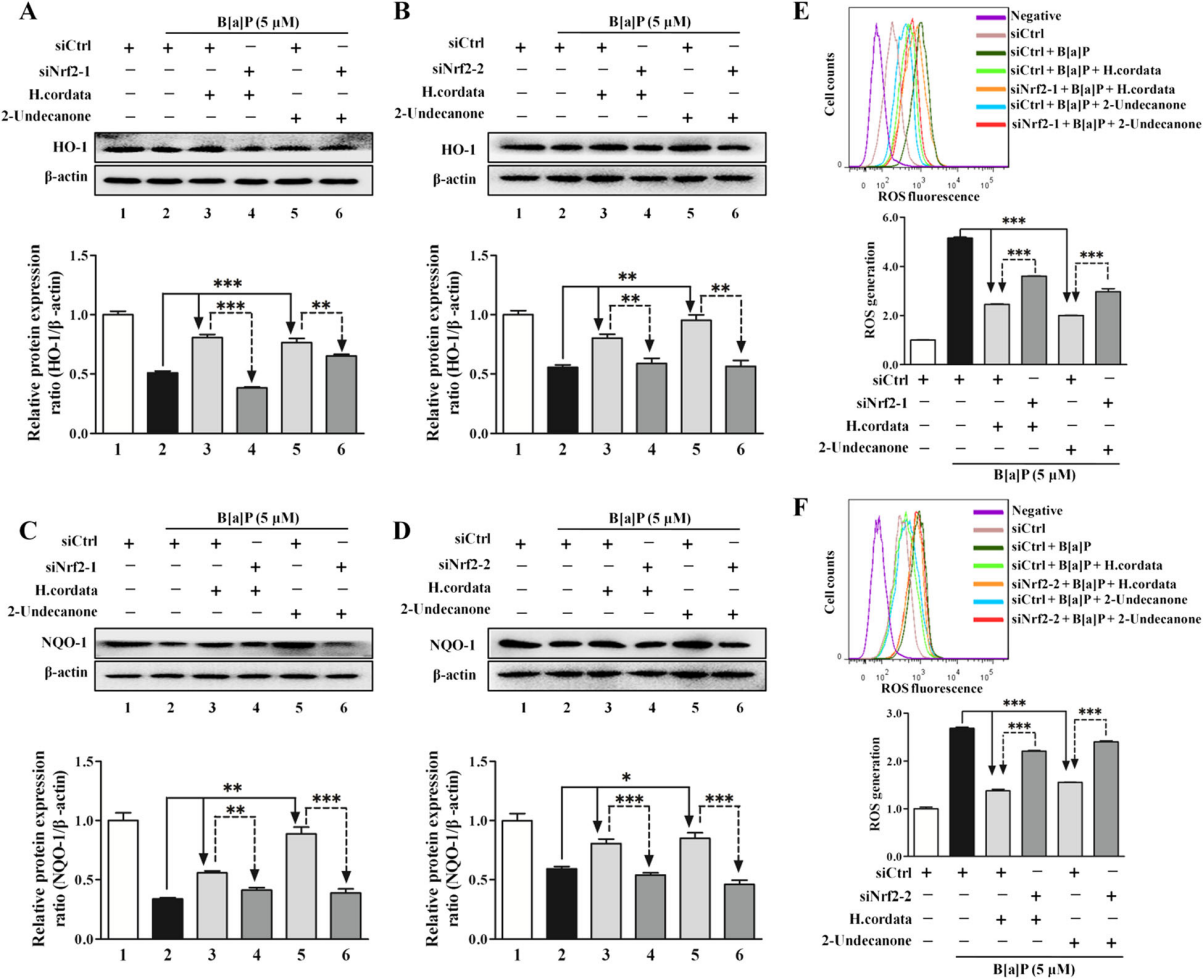
*H. cordata* and 2-undecanone induce Nrf2-mediated expression of HO-1 and NQO-1 to reduce B[a]P-induced ROS overproduction. Nrf2 expression was silenced in BEAS-2B cells by transfection with one of two Nrf2-specific siRNAs (siNrf2-1 or siNrf2-2). The protein levels were measured by using Western blot analysis. **a** and **b** The protein levels of HO-1 were measured in cells exposed to *H. cordata* (25 mg/mL) or 2-undecanone (50 μM) in the presence or absence of siNrf2-1 or siNrf2-2 for 48 h. **c** and **d**) The protein levels of NQO-1 were measured in cells subjected to the same treatments. **e** and **f** Intracellular ROS levels were detected in cells subjected to the described treatments by using flow cytometry. ^*^p < 0.05, ^**^p< 0.01, and ^***^p < 0.001 compared with the siCtrl group

To explore the mediating effect of Nrf2 on the protective effects of *H. cordata* and 2-undecanone against B[a]P-induced DNA damage and inflammation, Nrf2 expression was silenced in BEAS-2B cells by transfection with two Nrf2-specific siRNAs (siNrf2-1 and siNrf2-2). Cells transfected with siNrf2 showed significant reductions in Nrf2 expression compared to cells transfected with control siRNA (siCtrl) (Additional file 1: Figure S6, *p* < 0.001). Compared with cells transfected with siCtrl, cells transfected with siNrf2-1 or siNrf2-2 exhibited significant attenuations in the *H. cordata*- and 2-undecanone-enhanced protein levels of HO-1 (Fig. 8a and b, *p*< 0.01 or *p* < 0.001) and NQO-1 (Fig. 8c and d, *p* < 0.01 or *p* < 0.001). It was further observed that the reduction in B[a]P-induced ROS overproduction caused by *H. cordata* and 2-undecanone could be markedly attenuated by transfection with siNrf2-1 or siNrf2-2 (Fig. 8e and f, *p* < 0.001).

**Fig. 9:**
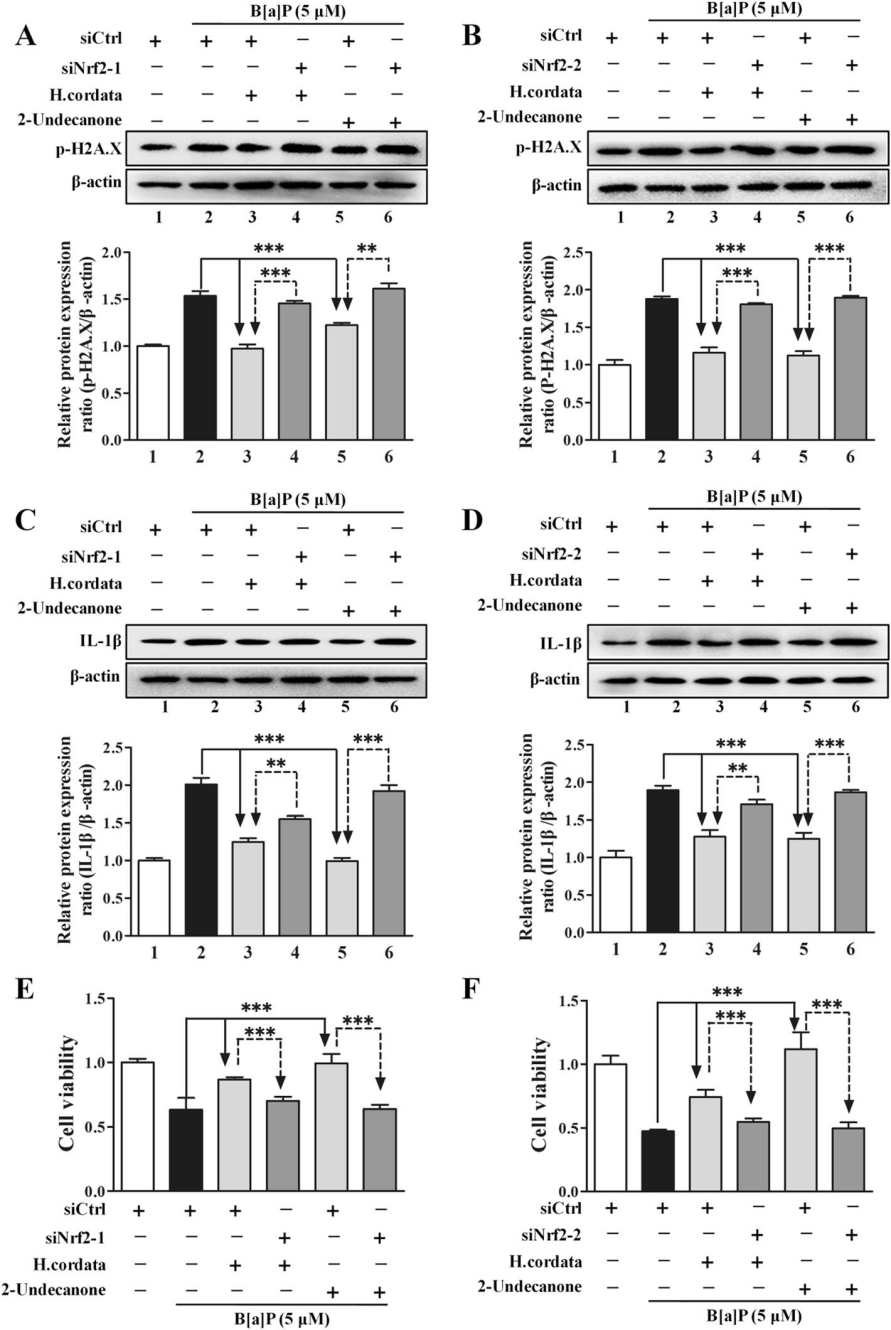
The Nrf2-HO-1/NQO-1 signaling pathway mediates the protective effects of *H. cordata* and 2-undecanone against B[a]P-induced DNA damage, inflammation and cytotoxicity. The protein levels were measured by using Western blot analysis. Cell viability was determined by MTT assay. **a** and **b** The protein levels of p-H2A.X were measured in cells exposed to *H. cordata* (25 mg/mL) or 2-undecanone (50 μM) in the presence or absence of siNrf2-1 or siNrf2-2 for 48 h. **c** and **d** The protein levels of IL-1β in cell culture supernatants were measured after treatment of cells with *H. cordata* (25 mg/mL) or 2-undecanone (50 μM) in the presence or absence of siNrf2-1 or siNrf2-2 for 24 h. **e** and **f** Cell viability was determined after treatment of cells with *H. cordata* (25 mg/mL) or 2-undecanone (50 μM) in the presence or absence of siNrf2-1 or siNrf2-2. ^**^p < 0.01 and ^***^p< 0.001 compared with the siCtrl group

In contrast to transfection with siCtrl, transfection with siNrf2-1 or siNrf2-2 markedly attenuated the *H. cordata* and 2-undecanone-mediated decreases in B[a]P-induced high p-H2A.X expression (Fig. 9a and b). In addition, the culture supernatants of cells transfected with siNrf2-1 or siNrf2-2 and incubated with *H. cordata* or 2-undecanone showed no changes or weak reductions in the B[a]P-induced high expression of IL-1β (Fig. 9c and d). Furthermore, the protective effects of *H. cordata* and 2-undecanone against B[a]P-induced cytotoxicity could be markedly attenuated by siNrf2 transfection (Fig. 9e and f).

## Discussion

Lung cancer is the most prevalent malignancy and is the leading cause of cancer death in men and the second leading cause of death in women [1]. B[a]P, which is mostly produced from cigarette smoking or car exhausts, acts as a potent carcinogen that leads to lung carcinogenesis [28]. Chemoprevention has been the most promising strategy to effectively decrease the incidence and mortality of lung cancer, especially for B[a]P-induced carcinogenesis in smoking patients [5, 6]. In the current study, we investigated the chemopreventive effects of *H. cordata*, a widely used herbal medicine with anti-inflammatory and antioxidative properties, against lung tumorigenesis and further clarified the underlying mechanisms. The results of this study will enhance understanding of the protective effect of *H. cordata* against B[a]P-stimulated lung tumorigenesis.

First, a B[a]P-stimulated lung adenocarcinoma animal model in A/J mice was successfully established to investigate the chemopreventive effects of *H. cordata* and its main bioactive compound 2-undecanone against lung cancer (Fig. 1a). A/J mice, which are highly susceptible to carcinogen-induced tumors, have been widely used as appropriate *in vivo* models to study the chemoprevention of lung carcinogenesis [5, 44]. The results showed that both *H. cordata* and 2-undecanone significantly reduced tumor numbers in B[a]P-treated mice in a dose-dependent manner compared to B[a]P alone (Fig. 1c). Moreover, the results obtained with the established B[a]P-treated BEAS-2B cell model showed that both *H.cordata* and 2-undecanone could effectively protect normal BEAS-2B cells from B[a]P-induced injury in a dose-dependent manner (Figs. 2 and 3). The combined results suggested that *H.cordata* could effectively prevent normal lung cells from B[a]P-initiated lung tumorigenesis and that 2-undecanone could be the bioactive compound responsible for this pharmacological activity.

Numerous chemopreventive agents have been developed, such as tamoxifen, raloxifene, finasteride, and celecoxib. However, the use of these agents for cancer prevention is restricted because of their severe side effects after long-term administration [4]. This issue clearly indicates that safe and efficacious agents are urgently needed for cancer prevention. Various herbal medicines and diet-derived natural products maybe candidates for this purpose. *H. cordata* is well known as a widely used medicinal herb with a variety of pharmacological functions [30, 31] and is also popularly consumed as a healthy vegetable in East Asia [32]. Thus, the toxicity of *H. cordata* was also determined *in vivo* and *in vitro* in this current study. Our data revealed no significant body weight differences between B[a]P control group A/J mice and treatment group A/J mice during the experiment (Fig. 1b). Moreover, no considerable differences were observed in the indexes of the heart, kidney, thymus, liver and spleen between the B[a]P control group and the treatment groups (Fig. 1d). In addition, incubation with *H.cordata* or 2-undecanone alone could not produce toxicity in normal BEAS-2B cells (Fig. 2a and b). These preliminary data implied that *H.cordata* could be a potential effective agent for the chemoprevention of lung carcinogenes is that does not cause severe systemic toxicity.

B[a]P-induced excessive production of ROS, which results in oxidative stress, can severely damage DNA structure and is a prerequisite for B[a]P-associated tumorigenesis [10–12]. It has been reported that DNA damage is significantly associated with increased lung cancer risk and is considered to be the primary cause of lung cancer development [45, 46]. The roles of DNA damage repair pathways in cancers have attracted widespread interest in the clinic and have been widely recognized as vital targets to improve lung cancer therapy [47]. Hence, the effects of *H. cordata* and 2-undecanone on B[a]P-induced DNA damage were further investigated. The results from the comet assay showed that *H. cordata* and 2-undecanone could notably reduce the fluorescence in migrated DNA and the tails of disrupted DNA fragments (Fig. 4a), suggesting that *H. cordata* and 2-undecanone could effectively reverse B[a]P-induced DNA damage. The levels of p-H2A.X, which is a verified marker for DNA double-strand breaks and is involved in the response to DNA damage [41–43], were determined *in vitro* and *in vivo*. The results showed that exposure to *H. cordata* or 2-undecanone significantly decreased the B[a]P-induced overexpression of p-H2A.X in a dose-dependent manner in the cells (Fig. 4b-d). p-H2A.X protein levels in the lung tissues of mice were further determined. It was observed that both *H. cordata* and 2-undecanone significantly downregulated p-H2A.X expression in B[a]P-treated mice compared with B[a]P treatment alone (Fig. 4e and f). Our results indicated that *H. cordata* and 2-undecanone could effectively reverse B[a]P-induced DNA damage *in vitro* and *in vivo*.

In addition to DNA damage, oxidative stress-induced inflammation is also a major contributor to lung cancer initiation and progression [13, 14]. Mature and biologically active IL-1β, which is generated from the inactive precursor cytokine pro-IL-1β and then secreted, is a critical regulator of the inflammatory response [48, 49]. IL-1β is involved in a variety of cellular activities, including cell proliferation, differentiation, and apoptosis [50]. Moreover, inflammation in the tumor microenvironment mediated by IL-1β is hypothesized to play a major role in cancer invasiveness, progression, and metastasis [51–53]. Studies on tumor immunosurveillance have proposed a strong relationship between lung cancer risk factors and alterations in IL-1β levels [54]. Anti-inflammatory therapy targeting the IL-1β innate immunity pathway can significantly reduce incident lung cancer and lung cancer mortality [55]. Hence, the effects of *H. cordata* and 2-undecanone on B[a]P-induced inflammation were also investigated. The results showed that treatment of cells with *H. cordata* or 2-undecanone significantly decreased the B[a]P-induced overexpression of IL-1β in the cell culture supernatants (Fig. 5 a, b, e and f). The same treatments also markedly decreased the high levels of pro-IL-1β and IL-1β induced by B[a]P in the cell lysates (Fig. 5a, c, d, e, g and h), suggesting that *H. cordata* and 2-undecanone could effectively reduce IL-1β secretion, thereby potentially reducing the inflammatory response. Furthermore, both *H. cordata* and 2-undecanone treatment could significantly downregulate the IL-1β levels in the plasma of the mice exposed to B[a]P (Fig. 5i). Our results implied that *H. cordata* and 2-undecanone could effectively reverse B[a]P-induced inflammation *in vitro* and *in vivo*.

Then, it was confirmed that incubation of cells with B[a]P could markedly increase intracellular ROS production (Fig. 6a) and notably enhance the expression of p-H2A.X (Fig. 6b and c), pro-IL-1β and IL-1β (Fig. 6d). However, NAC, a well-known ROS inhibitor, could markedly reverse the B[a]P-induced p-H2A.X overexpression (Fig. 6b and c). ROS reduction by NAC also significantly decreased the high levels of pro-IL-1β and IL-1β induced by B[a]P (Fig. 6d). From these results, it was confirmed that DNA damage and inflammation could be significantly reversed by reductions in intracellular ROS levels, suggesting that the B[a]P-induced overproduction of intracellular ROS could cause marked DNA damage and inflammation. Therefore, the impact of *H. cordata* and 2-undecanone on of intracellular ROS levels was further investigated. It was observed that both *H. cordata* and 2-undecanone treatment significantly diminished the intracellular ROS overproduction induced by B[a]P (Fig. 6e-g). The correlation between ROS levels and DNA damage or inflammation was analyzed by using Pearson analysis. The results showed that the decrease in ROS levels was closely and positively related to the inhibition of B[a]P-induced DNA damage (Additional file 1: Figure S4) or inflammation (Additional file 1: Figure S5) by *H. cordata* and 2-undecanone. Given all the results, it was speculated that *H. cordata* and 2-undecanone could protect cells against B[a]P-induced DNA damage or inflammation by reducing intracellular ROS overproduction.

**Fig. 10:**
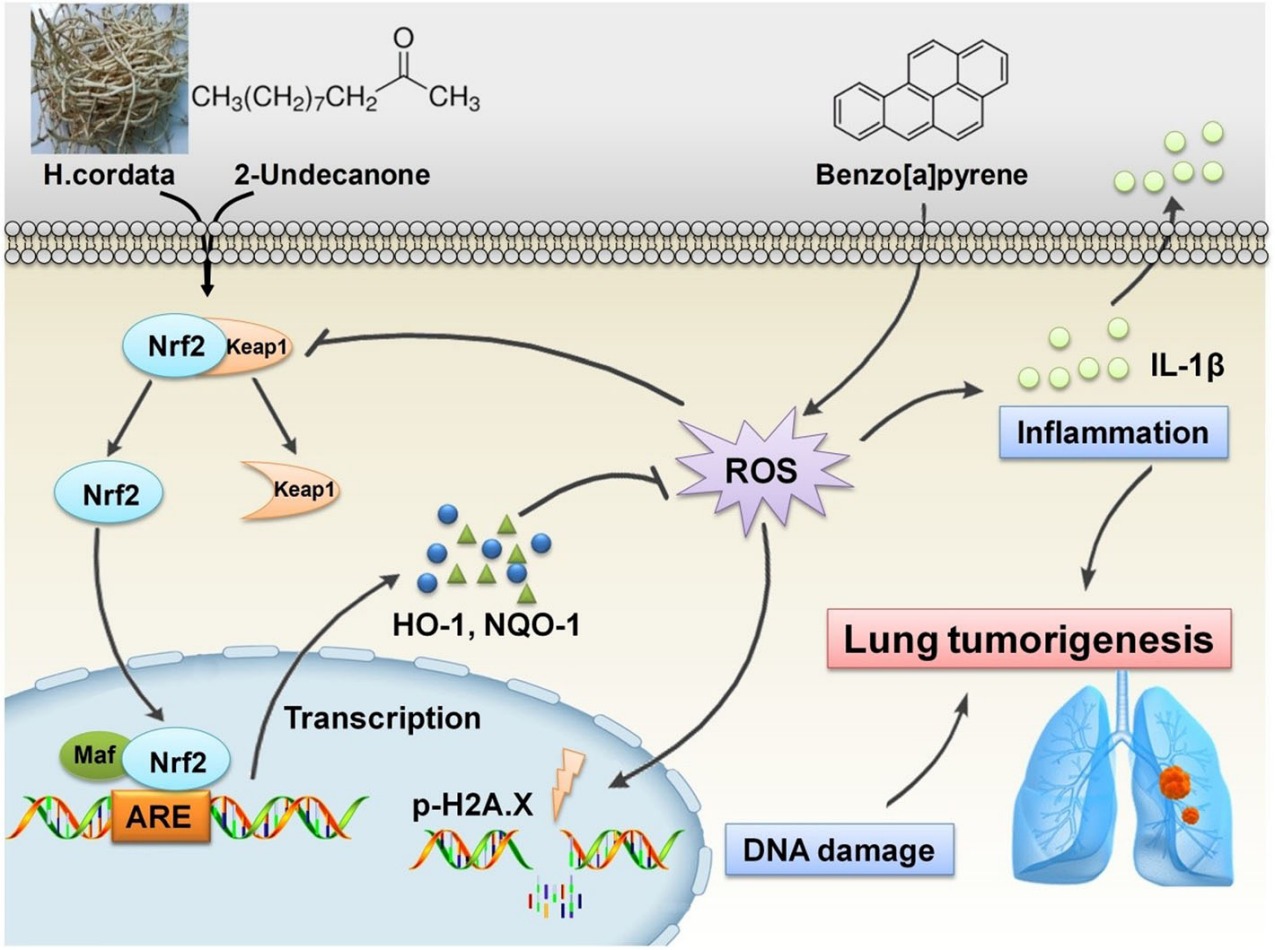
Schematic diagram of mechanism on this research. *H. cordata* and 2-undecanone could effectively activate the Nrf2 pathway to induce the expression of the antioxidative enzymes HO-1 and NQO-1 and thus to counteract intracellular ROS generation, thereby attenuating DNA damage and inflammation caused by B[a]P stimulation and playing a role in the chemoprevention of B[a]P-induced lung carcinogenesis

Nrf2 has been identified as a key regulator of the inducible expression of antioxidative enzymes, anti-inflammatory proteins and conjugation/detoxification proteins, such as NQO1 and HO-1 [21]. Upon oxidative stress caused by B[a]P or pharmacologic induction, the Nrf2 pathway could be rapidly activated to counteract intracellular ROS generation, thereby attenuating DNA damage and inflammation caused by B[a]P stimulation [21, 22]. The general cytoprotective effect of Nrf2 pathway activation has made it an attractive target for the chemoprevention of B[a]P-induced lung carcinogenesis. Hence, the modulatory effects of *H. cordata* and 2-undecanone on the Nrf2 pathway were further evaluated to clarify their chemopreventive functions against lung tumorigenesis. It was observed that both *H. cordata* and 2-undecanone significantly increased the protein levels of Nrf2, HO-1 and NQO-1 in whole-cell lysates (Fig. 7a) and resulted in significant increases in nuclear Nrf2 protein expression (Fig. 7b). The immunofluorescence results also showed that both *H. cordata* and 2-undecanone notably promoted the translocation of Nrf2 from the cytoplasm to the nucleus (Fig. 7c). Thus, it was implied that *H. cordata* and 2-undecanone could effectively activate the Nrf2 pathway to induce the expression of the antioxidative enzymes HO-1 and NQO-1. Then, it was found that B[a]P significantly decreased the protein levels of Nrf2, HO-1 and NQO-1 in whole-cell lysates (Fig. 7d) and decreased nuclear Nrf2 protein levels (Fig. 7e). In contrast, treatment with *H. cordata* and 2-undecanone markedly reversed the above-mentioned effects of B[a]P on the inhibition of Nrf2, HO-1 and NQO-1 expression (Fig. 7d and e). Moreover, both *H. cordata* and 2-undecanone treatment strikingly enhanced the expression of Nrf2, HO-1 and NQO-1 in a dose-dependent manner compared with B[a]P treatment in control mice (Fig. 7f-k). Then, to confirm the role of Nrf2 in the protective effects of *H. cordata* and 2-undecanone against B[a]P-induced DNA damage and inflammation, Nrf2 expression was silenced in BEAS-2B cells by transfection with siNrf2-1 or siNrf2-2. The data showed that transfection of cells with siNrf2-1 or siNrf2-2 significantly attenuated the *H. cordata*- and 2-undecanone-induced increases in the protein levels of HO-1 (Fig. 8a and b) and NQO-1 (Fig. 8c and d). The reductions in B[a]P-induced ROS overproduction mediated by *H. cordata* and 2-undecanone could also be markedly diminished by transfection of cells with siNrf2-1 or siNrf2-2 (Fig. 8e and f). Furthermore, the attenuating effects of *H. cordata* and 2-undecanone on B[a]P-induced p-H2A.X overexpression were markedly diminished by siNrf2 transfection (Fig. 9a and b). In addition, the culture supernatants of cells transfected with siNrf2-1 or siNrf2-2 and then treated with *H. cordata* or 2-undecanone showed no changes or weak reductions in the B[a]P-induced high levels of IL-1β (Fig. 9c and d). As expected, the protective effects of *H. cordata* and 2-undecanone against B[a]P-induced cytotoxicity were also markedly diminished by siNrf2 transfection (Fig. 9e and f). Based on the combined results *in vitro* and *in vivo*, it was speculated that *H. cordata* and 2-undecanone could effectively activate the Nrf2 pathway to induce the expression of the antioxidative enzymes HO-1 and NQO-1 and thus to counteract intracellular ROS generation, thereby attenuating DNA damage and inflammation causedby B[a]P stimulation and playing a role in the chemoprevention of B[a]P-induced lung carcinogenesis (Fig. 10).

## Conclusions

In conclusion, our studies have shown that *H. cordata* and its bioactive compound 2-undecanone can significantly reduce B[a]P-induced DNA damage and inflammation to prevent lung tumorigenesis by activating the Nrf2-HO-1/NQO-1 signaling pathway. The data from these well-controlled *in vitro* and *in vivo* studies indicate that *H. cordata* may exert beneficial effects against cigarette smoke-induced lung inflammation and oxidative DNA damage in the human body. Thus, *H. cordata* could be an effective candidate agent for the chemoprevention of lung cancer.

## Additional file

Additional file 1: **Figure S1.** The GC-FID chromatogram of standard compound 2-undecanone (A) and *H.cordata* water extract (B). **Figure S2.** Representative images showing haematoxylin and eosin staining of lung samples from the different groups. **Figure S3.** Effects of B[a]P on viability of BEAS-2B cells. Cell viability was examined using the MTT assay. The data represent the mean ± SD (n = 3). ^#^*p* < 0.05 and ^###^*p* < 0.001 compared with the control cells (given water). **Figure S4.** Pairwise correlation between reduction in B[a]P-induced ROS over production and decreased p-H2A.X protein levels ratio in BEAS-2B cells by *H. cordata* (A) and 2-undecanone (B). The correlations were analyzed by using Person analysis. **Figure S5.** Pairwise correlation between reduction in B[a]P-induced ROS over production and decreased protein levels of pro-IL-1β or IL-1β in BEAS-2B cells by *H. cordata* (A) and 2-undecanone (B). The correlations were analyzed by using Person analysis. **Figure S6.** The efficiency of Nrf2 silencing. Nrf2 expression was silenced in BEAS-2B cells by transfection of three Nrf2-specific siRNA (siNrf2-1, siNrf2-2 or siNrf2-3), respectively. The protein levels of Nrf2 were evaluated by using Western blot analysis. Data shown represent the mean ± SD (*n* = 3). ^***^p < 0.001 compared with the cells transfected with the control siRNA (siCtrl). siNrf2-1 and siNrf2-2 were selected for subsequent assays according to the efficiency of Nrf2 silencing. (DOCX 7901 kb)

## Data Availability

All data generated or analyzed during this study are included in this published article and its additional files.

## Abbreviations

B[a]P: benzo(a)pyrene
EdU: 5-Ethynyl-2’-deoxyuridine
ELISA: Enzyme-linked immunosorbent assay
*H. cordata*: *Houttuynia cordata* Thunb. (Saururaceae)
HO-1: heme oxygenase-1
IL-1β: interleukin-1β
MTT: 5-diphenyltetrazolium bromide
NAC: N-Acetyl-L-cysteine
NQO1: NAD(P)H: quinone oxidoreductase 1
Nrf2: nuclear factor E2-related factor-2
p-H2A.X: phosphorylated H2A.X
ROS: reactive oxygen species
tBHQ: tert-Butylhydroquinone
