# Supplementary material for: Houttuynia cordata Thunb. and its bioactive compound 2-undecanone significantly suppress benzo(a)pyrene-induced lung tumorigenesis by activating the Nrf2-HO-1/NQO-1 signaling pathway
Source: J Exp Clin Cancer Res. 2019 Jun 7;38:242. doi: 10.1186/s13046-019-1255-3 (PMC6556055; doi:10.1186/s13046-019-1255-3)

**Additional materials and methods**

**Standardization of *H.cordata* water extract by using gas chromatography with flame ionization detection (GC–FID)**

A total of 10 µLof ionconsisted of a GC-2010 Plus (Shimadzu Corporation, Japan) equipped with a flame ionization detector and a coldjet modulator. Chromatographic separation was carried out by using an Agilent J&W GC column (30 m × 0.250 mm × 0.250 μm, 100% dimethylpolysiloxane, Agilent Corporation, USA). Ultra-high-purity (99.999%) nitrogen was used as the carrier gas in a constant pressure mode. The inlet pressure was 100 kPa. An AOC-20i autosampler injected 2.0 μL of sample at a split ratio of 10:1 in a 270 °C inlet onto the column. The oven program was as follows: initial temperature, 120 °C (hold for 1 min); ramp at 10 °C/min up to 200 °C; and ramp at 50 °C/min to 250 °C (hold for 15 min). The temperature of the hydrogen flame ionization detector was 300 °C. The purge flow rate was 30 mL/min with a hydrogen flow rate of 90 ml/min and an air flow rate of 400 mL/min.

Several concentrations of standard solutions (from 0.164 to 84.2 µg/mL) spiked with 20 mg/ml 6-gingerol (IS) were directly analyzed with the GC–FID system. The calibration curve and correlation coefficients for 2-undecanone were obtained from the analytical results of GC–FID measurement. According to the calibration curve, the concentration of 2-undecanone was calculated. 2-Undecanone was found in *H.cordata* water extract at mean levels of 0.099 ± 0.011 mg/g (Supplementary Figure 1).

**Additional Figure legends**

**Additional Figure 1:** The GC-FID chromatogram of standard compound 2-undecanone (A) and *H.cordata* water extract (B).

**Additional Figure 2:** Representative images showing haematoxylin and eosin staining of lung samples from the different groups.

**Additional Figure 3:** Effects of B[a]P on viability of BEAS-2B cells. Cell viability was examined using the MTT assay. The data represent the mean ± SD (n = 3). ^#^*p* < 0.05 and ^###^*p* < 0.001 compared with the control cells (given water).

**Additional Figure 4:** Pairwise correlation between reduction in B[a]P-induced ROS over production and decreased p-H2A.X protein levels ratio in BEAS-2B cells by *H. cordata* (A) and 2-undecanone (B). The correlations were analyzed by using Person analysis.

**Additional Figure 5:** Pairwise correlation between reduction in B[a]P-induced ROS over production and decreased protein levels of pro-IL-1β or IL-1β in BEAS-2B cells by *H. cordata* (A) and 2-undecanone (B). The correlations were analyzed by using Person analysis.

**Additional Figure 6:** The efficiency of Nrf2 silencing. Nrf2 expression was silenced in BEAS-2B cells by transfection of three Nrf2-specific siRNA (siNrf2-1, siNrf2-2 or siNrf2-3), respectively. The protein levels of Nrf2 were evaluated by using Western blot analysis. Data shown represent the mean ± SD (*n* = 3). ^***^*p* < 0.001 compared with the cells transfected with the control siRNA (siCtrl). siNrf2-1 and siNrf2-2 were selected for subsequent assays according to the efficiency of Nrf2 silencing.

**Additional Figure 1**


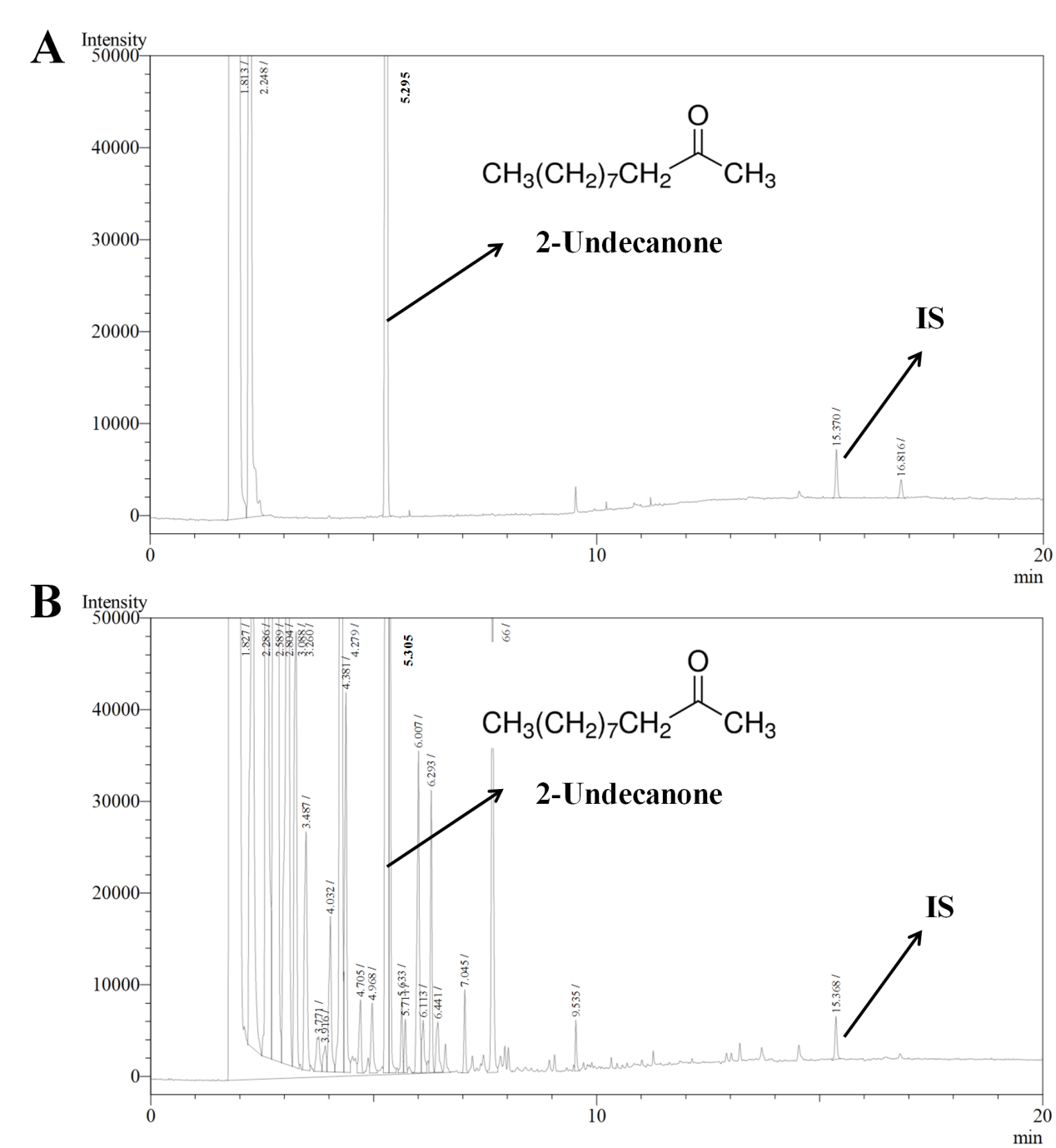


**Additional Figure 2**


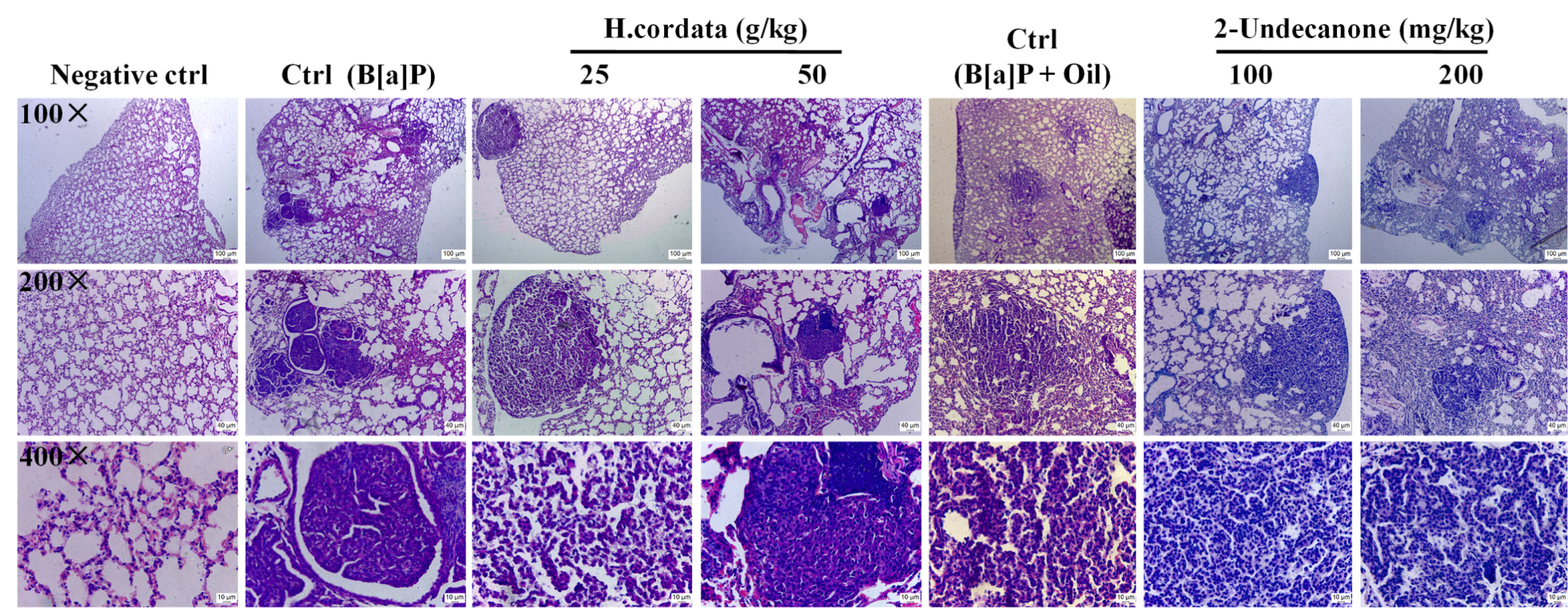


**Additional Figure 3**


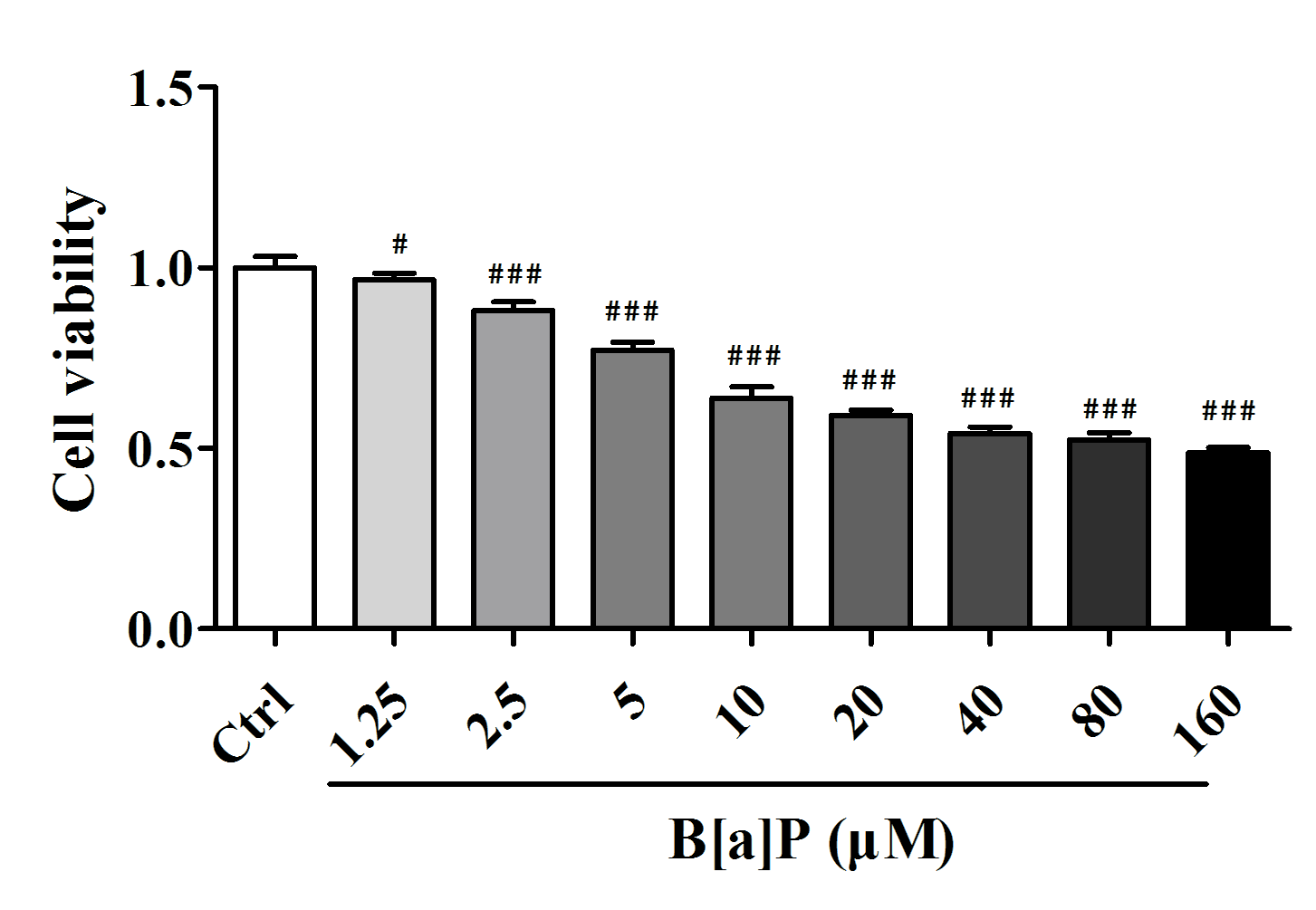


**Additional Figure 4**


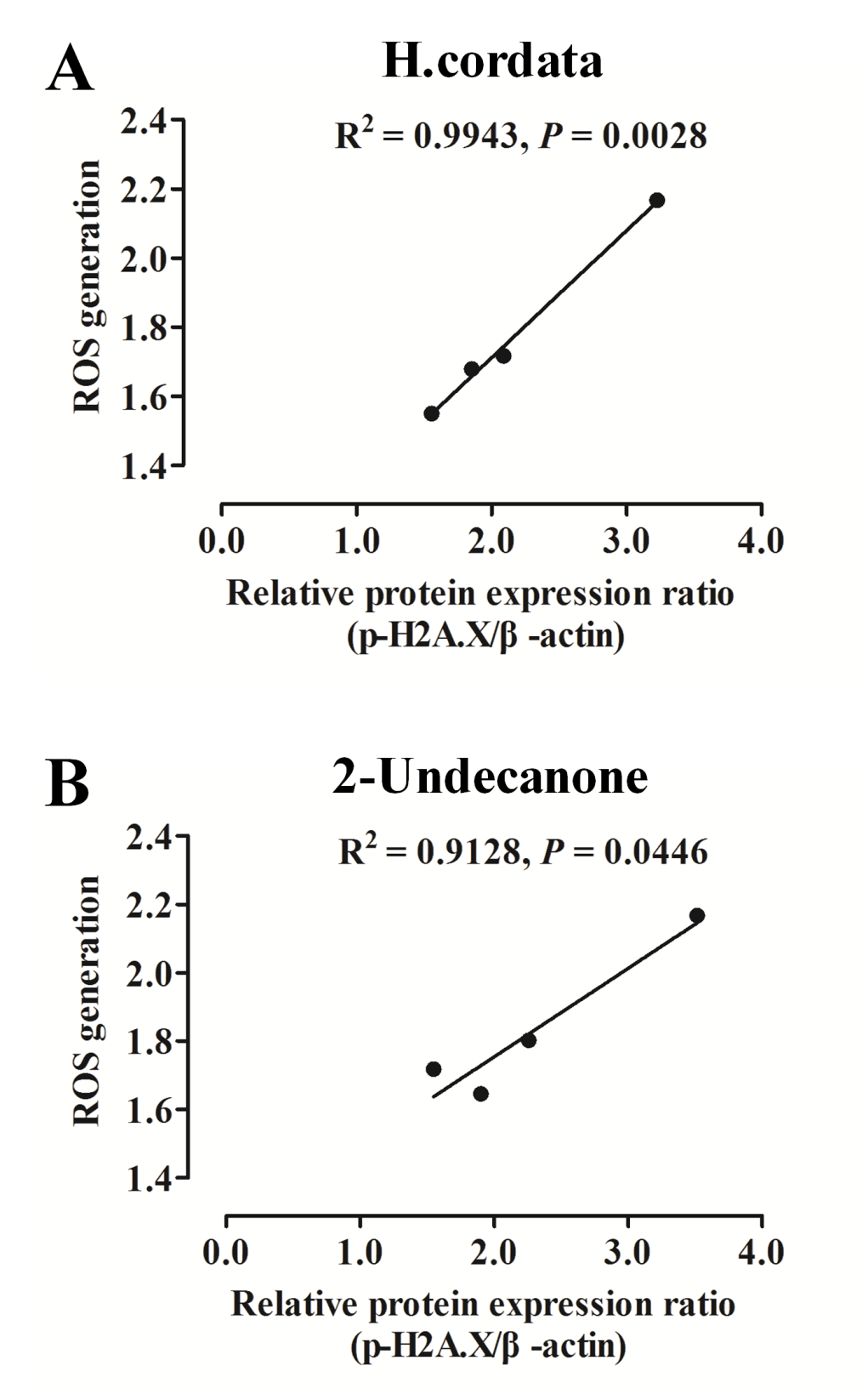


**Additional Figure 5**


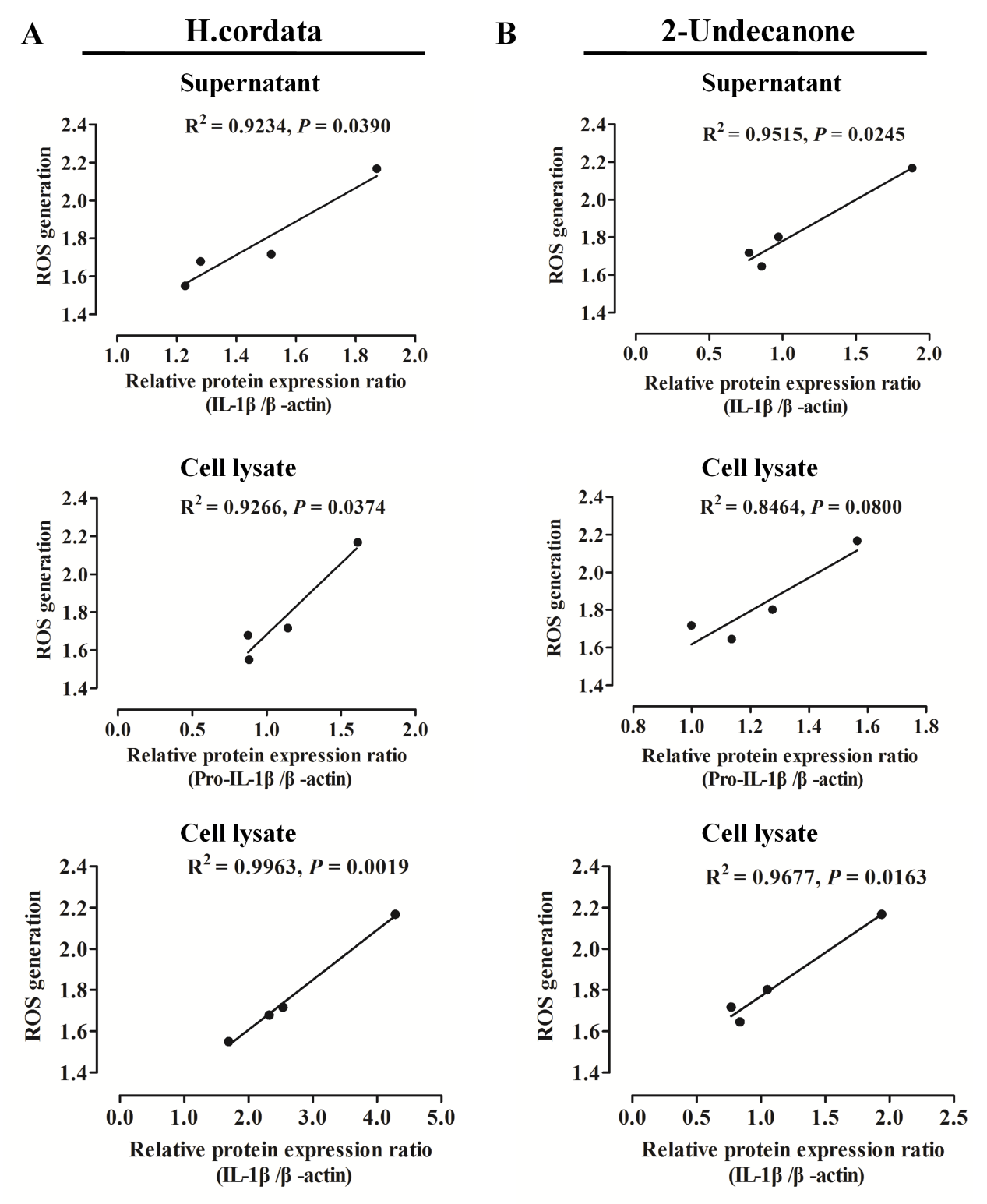


**Additional Figure 6**


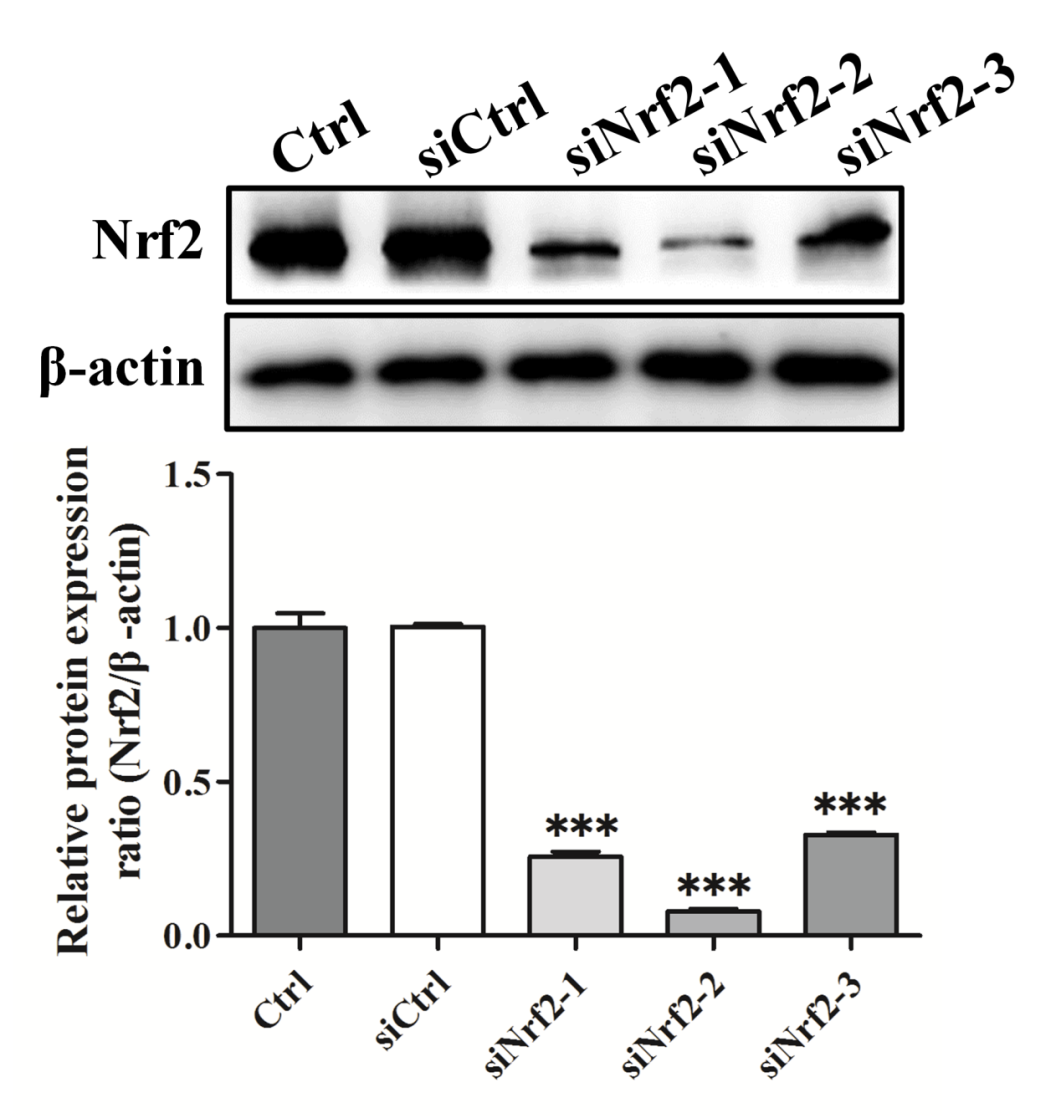

Supplement: Supplementary file 1 — Figure S1. The GC-FID chromatogram of standard compound 2-undecanone (A) and H.cordata water extract (B). Figure S2. Representative images showing haematoxylin and eosin staining of lung samples from the different groups. Figure S3. Effects of B[a]P on viability of BEAS-2B cells. Cell viability was examined using the MTT assay. The data represent the mean ± SD (n = 3). #p < 0.05 and ###p < 0.001 compared with the control cells (given water). Figure S4. Pairwise correlation between reduction in B[a]P-induced ROS over production and decreased p-H2A.X protein levels ratio in BEAS-2B cells by H. cordata (A) and 2-undecanone (B). The correlations were analyzed by using Person analysis. Figure S5. Pairwise correlation between reduction in B[a]P-induced ROS over production and decreased protein levels of pro-IL-1β or IL-1β in BEAS-2B cells by H. cordata (A) and 2-undecanone (B). The correlations were analyzed by using Person analysis. Figure S6. The efficiency of Nrf2 silencing. Nrf2 expression was silenced in BEAS-2B cells by transfection of three Nrf2-specific siRNA (siNrf2-1, siNrf2-2 or siNrf2-3), respectively. The protein levels of Nrf2 were evaluated by using Western blot analysis. Data shown represent the mean ± SD (n = 3). ***p < 0.001 compared with the cells transfected with the control siRNA (siCtrl). siNrf2-1 and siNrf2-2 were selected for subsequent assays according to the efficiency of Nrf2 silencing. (DOCX 7901 kb) [file 13046_2019_1255_MOESM1_ESM.docx]
